# Prospect certainty for data-driven models

**DOI:** 10.1038/s41598-025-89679-6

**Published:** 2025-03-10

**Authors:** Qais Yousef, Pu Li

**Affiliations:** Group of Process Optimization, Institute for Automation and Systems Engineering, Technische Universität Ilmenau, P.O. Box 100565, 98684 Ilmenau, Germany

**Keywords:** Data-driven model, Distributional change, Prospect theory, Uncertainty, Computer science, Computational science

## Abstract

The inherent nature of uncertainty in the inputs of data-driven models can lead to incorrect outputs. Such outcomes are difficult to ascertain due to the lack of reference data during the deployment, which hinders their acceptance in practical applications. This highlights the need to evaluate the degree of certainty of the output of a model to improve its robustness. In this paper, we present a new method for quantifying the output certainty of data-driven models, considering changing probability distributions of input data during the deployment. We achieve this by introducing the concept of logit masking to mitigate the deterministic nature of the model and build multiple alternatives for each output logit. Then, we propose a weighted probability function to provide an initial insight into the certainty of these alternatives. Moreover, we define a behavior function to describe the degree to which these alternatives affect the output distribution pattern. By combining these weights and behaviors, we determine the prospect certainty of these variants and finally choose the one with the highest certainty as the refined output of the model. Experimental results on benchmark and real-world datasets show that our proposed method outperforms state-of-the-art techniques in determining output certainty for data-driven models.

## Introduction

Uncertainty in data-driven models significantly degrades their reliability in real-world applications such as autonomous driving, medical imaging, and robotic surgery. During the training phase, the model utilizes data from a known distribution to optimize its parameters, minimizing the error between its predicted outputs and the target values. This process enhances the model’s predictive accuracy and increases the certainty of its outputs when processing inputs from this familiar distribution.

However, due to the stochastic nature of the input data, the optimizer may not always converge to a global optimum. Instead, it often settles into local minima or saddle points, introducing an intrinsic source of uncertainty that affects the accuracy of the model outputs during its deployment.

When deployed, the trained model processes new input data using the same learned parameters, even though the new data often originates from an unknown and unpredictable distribution. The discrepancy between the training and deployment data distributions introduces an additional layer of uncertainty. Consequently, it is challenging for the trained model to maintain the same level of output accuracy as it achieved during the training phase.

Once a model is trained, its input–output relation is deterministic, i.e., it generates an output for every input. Considering the inherent uncertainties within such models and their inputs, it is crucial to quantify the certainty of the output they produce to provide highly robust and reliable predictions, thereby enhancing their trustworthiness in real-world settings.

Uncertainty in data-driven models has been extensively studied primarily focusing on Bayesian-based methods^1–4^. These approaches utilize a prior over the weights to capture the posterior distribution and evaluate the uncertainty by estimating expected predictions through stochastic sampling, albeit at a significant computational cost. Despite their effectiveness, Bayesian techniques often lead to overconfidence and reliance on biased approximations. Alternatives like ensemble models^5,6^ and single model approaches^7–11^, paired with active learning^12,13^, enhance prediction accuracy but remain computationally intensive. Evidential learning-based bypass sampling^14,15^ estimates uncertainty directly, yet faces challenges with intractable posterior distributions, as their complexity increases. Although the complexity problem is successfully addressed by deterministic uncertainty^16–21^ and stochastic regularization^22–24^ methods, they cannot guarantee to perform effectively in real-world settings. Overall, existing methods fail to account for distributional changes during the deployment of the model or explicitly quantify the certainty of the outputs.

This paper introduces a novel approach, coined *prospect certainty,* to quantify the certainty of the outputs in data-driven models to enhance their robustness across various input distributions. For instance, consider a model trained to classify brain cancer using medical images obtained from a specific type of sensor. When this model is deployed in a new environment with images from a different sensor type, it typically generates outputs without accounting for the sensor change which affects the distribution of the input images. Nevertheless, utilizing the prospect certainty method proposed in this paper, the model can provide a certainty degree of its output along with a refined output value that accounts for the distributional changes of the input data. This enhancement enables the model to be more robust and reliable in real-world applications.

We achieve this by initially introducing variability into the deterministic nature of a trained model by *logit masking*. As a result, we generate new alternatives for some selected output nodes (logits) by connecting each mask differently to the previous layer to increase their disparity. To estimate the initial certainty of each alternative, we introduce a *weighted probability* function. This function is calculated based on the disparity between the logits and their masks, and their deviation from the mean.

Next, we assess each logit’s behavior in response to the changes in the input distribution by a *behavior function*. With this function, we evaluate the impact of each logit on the pattern of the output, measured by the Wasserstein distance between the distributions of the model output and the training labels. Finally, we integrate the weighted probability for the nodes with their corresponding output of the behavior function and employ the Kahneman and Tversky’s prospect theory^25,26^ to quantify the prospect certainty value for each logit. The logit with the highest prospect certainty is selected as a refined output of the model.

As a result, the extended model can provide a degree of certainty in prospect with its attitude toward different sources of uncertainty and its behavior toward input distributional changes. Moreover, this method improves the accuracy of the model’s output. Therefore, the proposed prospect certainty contributes to enhancing the reliability of the model when it is deployed in a variable environment where the distribution of input data changes randomly, as detailed in^27^. In addition, it enhances the reliability of the model when it confronts an unfamiliar input that is expected to lead to an output beyond the scope of its trained ground truth. Furthermore, our method provides a means to measure the direct influence of the certainty function on the output error of the trained model.

The remainder of the paper is structured as follows. In Sect. “Material and methods”, we present the fundamentals of the proposed approach. After describing the problem, a method to construct the prospect certainty is developed. In Sect. “Experiments and results discussion”, the results of case studies including benchmark experiments and the detection of pedestrian attention based on a real dataset are presented to demonstrate the effectiveness of the proposed approach. Sect. “Conclusions” concludes the paper and points out relevant future works.

## Materials and methods

In this section, we introduce the *prospect certainty* approach by first defining the problem of input distribution shift in data-driven models. We then detail the steps of our solution and demonstrate how it effectively addresses the defined problem. It is worth noting that, in this paper, the term *uncertainty* encompasses all forms of uncertainty that a model may generally encounter. Furthermore, the term *node* is used to denote either an original logit or a generated mask.

### Code availability

A corresponding code that demonstrates the implementation of the proposed method is provided in^28^ facilitating a deeper understanding of the deployment of our approach to quantifying the prospect certainty of the output of data-driven models.

### Problem description

A general form of a data-driven model $f$ can be expressed as^29^,

1 $$\widehat{{\varvec{u}}}=f\left({\varvec{x}},{\varvec{\theta}}\right)$$

where, $\widehat{{\varvec{u}}}$ is the output (logit) vector, ${\varvec{x}}$ is the input vector, and ${\varvec{\theta}}$ is the (weight) parameter set. The output of this model can be written as^29^,

2 $$\widehat{{\varvec{y}}}=g(\widehat{{\varvec{u}}})$$

where $g(.)$ is an activation function. The model $f$ is trained on $T$ paired samples ${\left({{\varvec{x}}}_{t}^{s},{{\varvec{y}}}_{t}^{s}\right)}_{t=1}^{T}.$ During the training phase the input vector ${{\varvec{x}}}^{s}$ is drawn from a given distribution ${p}_{x}^{s}$, which is called here the *source distribution*. Moreover, the output label vector ${{\varvec{y}}}^{s}$ is drawn from a known source distribution ${p}_{y}^{s}$. The training phase determines the optimal parameters ${{\varvec{\theta}}}^{*}$. Since the model generates output for each input data, i.e. $:{\varvec{x}}\to {\varvec{y}}$ , two possible uncertainty sources in the model output can arise. i) During model deployment, a new input sample ${{\varvec{x}}}^{t}$ drawn from an unknown *(target) distribution* ${p}_{x}^{t}$ often appears. Although ${{\varvec{x}}}^{t}\sim {p}_{x}^{t}$ should yield the output ${\widehat{{\varvec{y}}}}^{t}$ that falls within the source distribution of the labels ${p}_{y}^{s}$, the model $f$ with its learned weights ${{\varvec{\theta}}}^{*}$ may produce a divergent output $p\left(g\left(f({{\varvec{x}}}^{t},{{\varvec{\theta}}}^{*})\right)\right)\not\approx {p}_{y}^{s}$. This is because ${{\varvec{\theta}}}^{*}$ is optimized for the distribution ${p}_{x}^{s}$. For example, if the model is trained on medical images collected using a specific type of scanner, it is unlikely to produce accurate outputs when processing images from a different scanner. ii) When deploying $f$, an irrelevant input sample ${{\varvec{x}}}^{irr}\sim {p}_{x}^{irr}$ might appear, which does not belong to the feature space learned by $f$. Despite this, while $f$ must produce an output, it processes ${{\varvec{x}}}^{irr}$ and generates an incoherent output value that could fall within the source distribution of the originally trained labels, i.e. $p\left(g\left(f({{\varvec{x}}}^{irr},{{\varvec{\theta}}}^{*})\right)\right)\approx {p}_{y}^{s}$. For instance, if the model is trained to classify images of dogs and cats, it will incorrectly classify an image of a boat as either a dog or a cat.

Given these challenges, it is expected that the model should have the capability to explicitly generate a quantified certainty degree in prospect with its behavior and response to distributional changes.

### Proposed solution

In this sub-section, we detail the proposed method for explicitly quantifying the certainty of the logits in a data-driven model. This method enhances the accuracy, robustness, and reliability of the model by selecting the output with the highest certainty. Our solution is based on the following two assumptions.

#### Assumption 1:

Assume that a model $f$ is trained on ${\left({{\varvec{x}}}_{t}^{s},{{\varvec{y}}}_{t}^{s}\right)}_{t=1}^{T}\sim \left({p}_{x}^{s},{p}_{y}^{s}\right)$, then, if $p\left(g\left(f\left({{\varvec{x}}}^{t,1},{{\varvec{\theta}}}^{*}\right)\right)\right)={p}_{y}^{s}$ and $p\left(g\left(f({{\varvec{x}}}^{t,2},{{\varvec{\theta}}}^{*})\right)\right)\ne {p}_{y}^{s}$ , where $\left\{{{\varvec{x}}}^{t,1},{{\varvec{x}}}^{t,2}\right\}\sim {p}_{x}^{t}$ and ${p}_{x}^{t}\ne {p}^{s}$. Then, the Wasserstein distance between the distributions of the output and the training labels are as follows; ${\text{W}}_{2}\left({\widehat{{\varvec{y}}}}^{t,1},{{\varvec{y}}}^{s}\right)\ll {\text{W}}_{2}\left({\widehat{{\varvec{y}}}}^{t,2},{{\varvec{y}}}^{s}\right)$. There should be a certainty function $\Omega$, such that $\Omega \left({\widehat{{\varvec{y}}}}^{t,1}\right)\gg \Omega \left({\widehat{{\varvec{y}}}}^{t,2}\right),$ to reflect the accuracy variation.

#### Assumption 2:

If $p\left(g\left(f\left({{\varvec{x}}}^{t},{{\varvec{\theta}}}^{*}\right)\right)\right)={p}_{y}^{s}$, and $p\left(g\left(f\left({{\varvec{x}}}^{irr},{{\varvec{\theta}}}^{*}\right)\right)\right)={p}_{y}^{s}$, where ${{\varvec{x}}}^{t}\sim {p}_{x}^{t}$, and ${{\varvec{x}}}^{irr}\sim {p}_{x}^{irr}; {p}_{x}^{irr}\ne {p}_{x}^{t}$ is out of the feature space of ${{\varvec{x}}}^{s}$ and should produce an irrelevant output. Then ${\text{W}}_{2}\left({\widehat{{\varvec{y}}}}^{t},{{\varvec{y}}}^{s}\right)\approx {\text{W}}_{2}\left({\widehat{{\varvec{y}}}}^{irr},{{\varvec{y}}}^{s}\right)$. There should be a certainty function $\Omega$, such that $\Omega \left({\widehat{{\varvec{y}}}}^{t}\right)\gg \Omega \left({\widehat{{\varvec{y}}}}^{irr}\right)$, to give reliability and robustness to the model.

To quantify the certainty of the outputs for a data-driven model, aligned with Assumptions 1 and 2, the proposed prospect certainty should explicitly reflect both the distribution of the model output and the relevance of its input to the feature space. Accordingly, our solution comprises the following four steps.

#### Logit masking

**Fig. 1:**
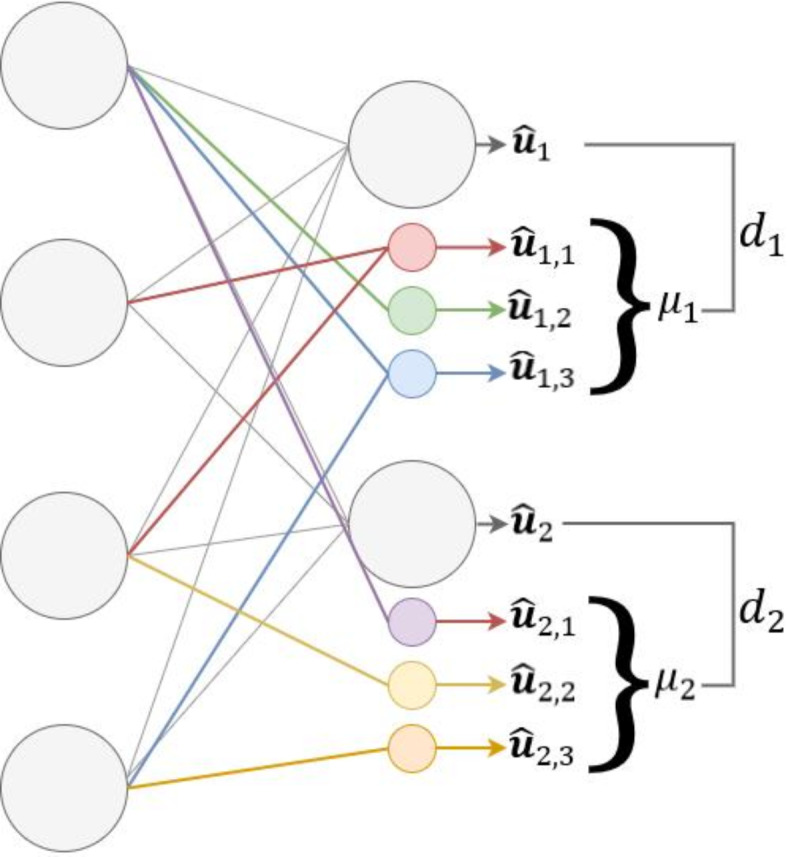
Method of masking and ratio:$\it {{N}_{1}^{M} =3, {N}_{2}^{M}=3, {R}_{1}=\left\{0.5, 0.25, 0.5\right\} , {R}_{2}=\left\{0.25, 0.25, 0.25\right\}}$.

Initially, it is crucial to ensure that the model accounts for distributional changes in the input when generating outputs. To achieve this, we introduce variability into the deterministic nature of the model by allowing for more alternatives for each output logit. We call this method *logit masking*, where we generate ${N}_{i}^{M}$ masks for a selected logit ${\widehat{u}}_{i}\in {\left\{{\widehat{u}}_{i}\right\}}_{i=1}^{{N}^{L}}$ of the output layer. For example, the logit ${\widehat{u}}_{i}$ may have masks ${\left\{{\widehat{u}}_{i,j}\right\}}_{j=1}^{{N}_{i}^{M}}$ of the same type as the original logit. Unlike the original logit ${\widehat{u}}_{i}$, which may be fully connected to the nodes of the previous layer, these masks are not fully connected, as illustrated in Fig. 1. The mask-wise random connection ratio vector ${R}_{i}={\left\{{r}_{i,j}\right\}}_{j=1}^{{N}_{i}^{M}}$ indicates the number of random connections for each mask with the neurons of the previous layer. This random connection increases the degree of dispersion of the output of the masks, enabling them to exhibit variability in their behavior. The number of masks ${N}^{M}$ and the corresponding ratios $R$ are hyperparameters set by the user.

For example, as illustrated in Fig. 1, consider a network with two logits in its output layer and four nodes in the preceding layer. If we design the model structure with ${N}_{1}^{M}=3$, it means generating three masks for the first logit. These masks are connected randomly to nodes in the previous layer based on specified ratios. For instance, with ${R}_{1}=\left\{0.5, 0.25, 0.5\right\}$, the first mask connects to 50% of the nodes (2 nodes). The second mask connects to 25% of the nodes (1 node), and the third mask connects to 50% of the nodes (2 nodes), all chosen randomly. This results in varied connections for each mask in terms of node indices and amount.

Similarly, setting ${N}_{2}^{M}=3$ generates three masks for the second logit. With ${R}_{2}=\left\{0.25, 0.25, 0.25\right\}$, each mask connects to 25% (1 node) of the previous layer, selected randomly and distinctly.

#### Weighted probability

**Fig. 2:**
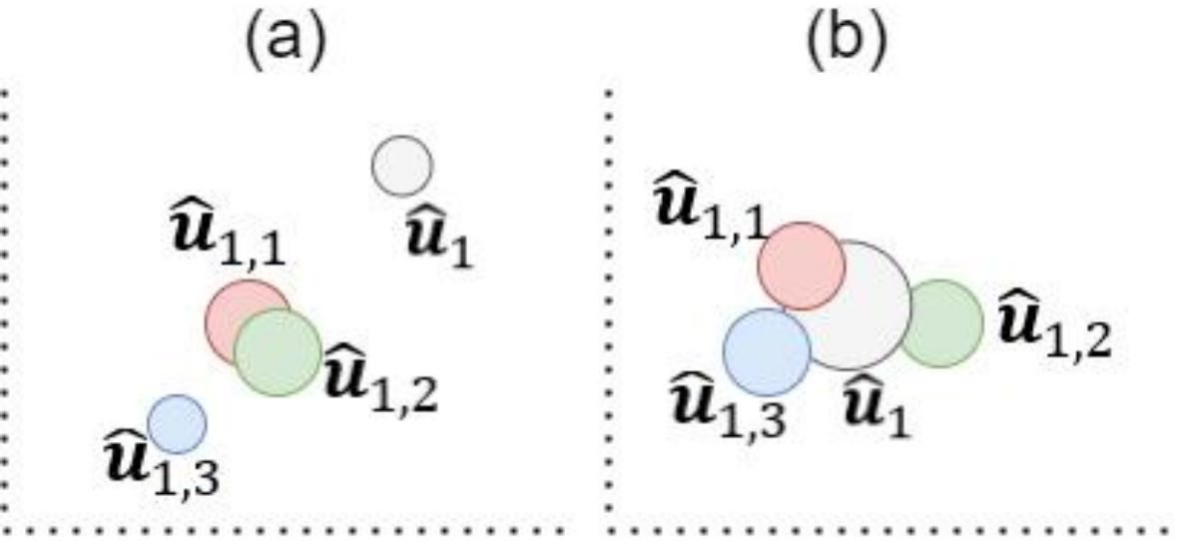
Illustration of weighted probability ${\text{Pr}}^{w}$, which is intricately linked to the variability of the node’s outputs. Here, the node’s ${Pr}^{w}$ is illustrated by the diameter of its circle. In (**a**) the masks’ values $\it {{\widehat{\varvec{u}}}_{1,1}, {t}}$ and $\it {\widehat{\varvec{u}}}_{{1,2},{t}}$, which are near the mean, exhibit higher ${{Pr}}^{w}$. In (**b**), the original logit and its masks display values centered near the mean, thus the original value of the logit $\it {\widehat{\varvec{u}}}_{1,{t}}$ shows the highest ${{Pr}}^{w}$.

To provide an initial insight into the certainty of the logit output, considering its masks, we introduce the concept of *weighted probability* for the nodes. Each logit mask is associated with a different number of connections to the previous layer’s nodes, resulting in slightly different values for the original logit. To select the output and calculate the probability of this logit, we need to consider the following important criteria: the number of occurrences of each value produced by the logit and its masks, the sparsity of these values, and the variance between the mean of the masks’ values and the original logit’s value, as illustrated in Fig. 2. These criteria should be weighted appropriately to reflect their correct contribution to the logit’s output.

Step 1: We calculate the probability for each original logit and its associated masks individually. This is done by summing the number of occurrences of the node output within the others corresponding to the same logit. We then divide this number by the total number of associated masks plus one (accounting for the original logit). For example, the probability of the output of logit ${\widehat{u}}_{i,t}$ and each of its masks ${\left\{{\widehat{u}}_{i,j,t}\right\}}_{j=1}^{{N}_{i}^{M}}$ is calculated as follows:

3 $${\text{Pr}}_{t}\left({\widehat{u}}_{t}\right)=\frac{count\left({\widehat{u}}_{t} , \left[{\left\{{\widehat{u}}_{i,j,t}\right\}}_{j=1}^{{N}_{i}^{M}}, {\widehat{u}}_{i,t}\right]\right) }{{N}_{i}^{M}+1}$$

where ${\widehat{u}}_{t}$ can be substituted by the node for which the number of occurrences of its value needs to be determined. The term $count\left({\widehat{u}}_{t} , \left[{\left\{{\widehat{u}}_{i,j,t}\right\}}_{j=1}^{{N}_{i}^{M}},{\widehat{u}}_{i,t}\right]\right)$ is interpreted as counting the number of occurrences of the node value ${\widehat{u}}_{t}$ within the list that includes the logit value ${\widehat{u}}_{i,t}$ and all its corresponding masks ${\left\{{\widehat{u}}_{i,j,t}\right\}}_{j=1}^{{N}_{i}^{M}}$. This form of probability implies equal significance for the original logit and each of its masks. However, the probability of the original logit should not be equally weighted with its corresponding masks, since the original logit has more connections to the previous layer. This could lead to a more robust value but also to overfitting. Additionally, the probability should be affected by the inputs and model uncertainties, reflecting the model’s response to them.

Step 2: To address these requirements, the logit and each of its corresponding masks should be weighted in a way to ensure the reflection of their disparity level (see Fig. 2). Such as, when the masks are relatively scattered, indicating the uncertainty, the weight of the original logit should increase. This disparity level can be measured by the absolute difference $d$ between the node’s value and the mean of all corresponding masks $\mu \left({\left\{{\widehat{u}}_{i,j,t}\right\}}_{j=1}^{{N}_{i}^{M}}\right)$. Conversely, when the masks are relatively converged, suggesting certainty, the weight of the original logit should decrease relative to its masks. Since the overall influence of the logit should be, to some extent, higher than its masks; if the logit value is relatively close to the mean of its masks (also calculated by $d$), its weight should be higher, even compared to a mask with the same distance from the mean. To ensure these criteria, we define the weights for the logit and each of its masks as follows,

4 $${w}_{i,t} = \frac{1}{{\ln \left( {\frac{{\left| {\hat{u}_{i,t} - \mu \left( {\left\{ {\hat{u}_{i,j,t} } \right\}_{j = 1}^{{N_{i}^{M} }} } \right)} \right|}}{d} + e + \epsilon } \right)}}$$

5 $${w}_{i,j,t} = \frac{1}{{\ln \left( {\frac{{\left| {\hat{u}_{i,j,t} - \mu \left( {\left\{ {\hat{u}_{i,j,t} } \right\}_{j = 1}^{{N_{i}^{M} }} } \right)} \right|}}{d} + e + \epsilon + s} \right)}}$$

where ${w}_{i,t}$ and ${w}_{i,j,t}$ are used to calculate the weight of the logit and each of its masks, respectively. $e$ is Euler’s number to ensure that the natural log is always positive. $\epsilon ={10}^{-5}$ is a small positive constant to avoid the division by zero. $s={10}^{-3}$, is a small bias constant to give slightly more influence to the logit. The incorporation of the natural logarithm into the weight calculation provides a more balanced and interpretable distribution of the weights. As a result, this approach manages outliers effectively, simplifies complex relationships, and ensures that the weights remain positive and proportional. The definitions in (4) and (5) are empirically derived to satisfy the functionality of the weighted probability function. Noting that, to the best of our knowledge, no standard or widely recognized approach exists in the open literature that meets these specific requirements. The proof is provided in the supplementary material demonstrating that the definitions meet the requirements of the weighted probability function.

Step 3: The weighted probability is calculated by multiplying the weight of each node and its probability. For instance, the weighted probability of the logit ${\widehat{u}}_{i,t}$ and each of its masks is,

6 $${\text{Pr}}_{t}^{w}({\widehat{u}}_{t},{w}_{t})={w}_{t} {\text{Pr}}_{t}\left({\widehat{u}}_{t}\right)$$

To ensure that each node corresponding to the same logit has a weighted probability relative to the other nodes within the same logit, we normalize their respective probabilities as follows,

7 $${\widehat{\text{Pr}}}_{t}^{w}\left({\widehat{u}}_{t},{w}_{t}\right)=\frac{{\text{Pr}}_{t}^{w}({\widehat{u}}_{t},{w}_{t})}{{\text{Pr}}_{i,t}^{w}\left({\widehat{u}}_{i,t},{w}_{i,t}\right) + \sum_{j=1}^{{j={N}{M}_{i}}}{\text{Pr}}_{i,j,t}^{w}({\widehat{u}}_{i,j,t},{w}_{i,j,t})}$$

where ${\widehat{u}}_{t}$ and ${w}_{t}$ can be substituted with the node to be evaluated and its corresponding weight. The formulation (7) ensures that ${\widehat{\text{Pr}}}_{i,t}^{w}\left({\widehat{u}}_{i,t},{w}_{i,t}\right)+\sum_{j=1}^{{j={N}{M}_{i}}}{\widehat{\text{Pr}}}_{i,j,t}^{w}({\widehat{u}}_{i,j,t},{w}_{i,j,t})=1$.

Step 4: For data-driven models with classification tasks, the refined logit value ${\widehat{u}}_{i,t}^{*}$ is taken by selecting the value of the node (original logit or its masks) with the highest weighted probability. If more than one node shows similar weighted probability, the average of their values is selected, although it rarely happens. For regression tasks, the refined logit value is selected by the higher prospect certainty of its nodes, as detailed in Sect. “Prospect certainty”.

The weighted probability offers an initial measure of the certainty associated with the logit output, significantly enhancing its accuracy. However, this measure alone is inadequate for comprehensively determining the certainty level of the original logit, since it fails to account for the impact of distributional changes in input data on the model output. To address this limitation, we introduce a behavior function that is calculated independently for each logit to provide a more robust assessment of the prospect certainty.

#### Behavior function

**Fig. 3:**
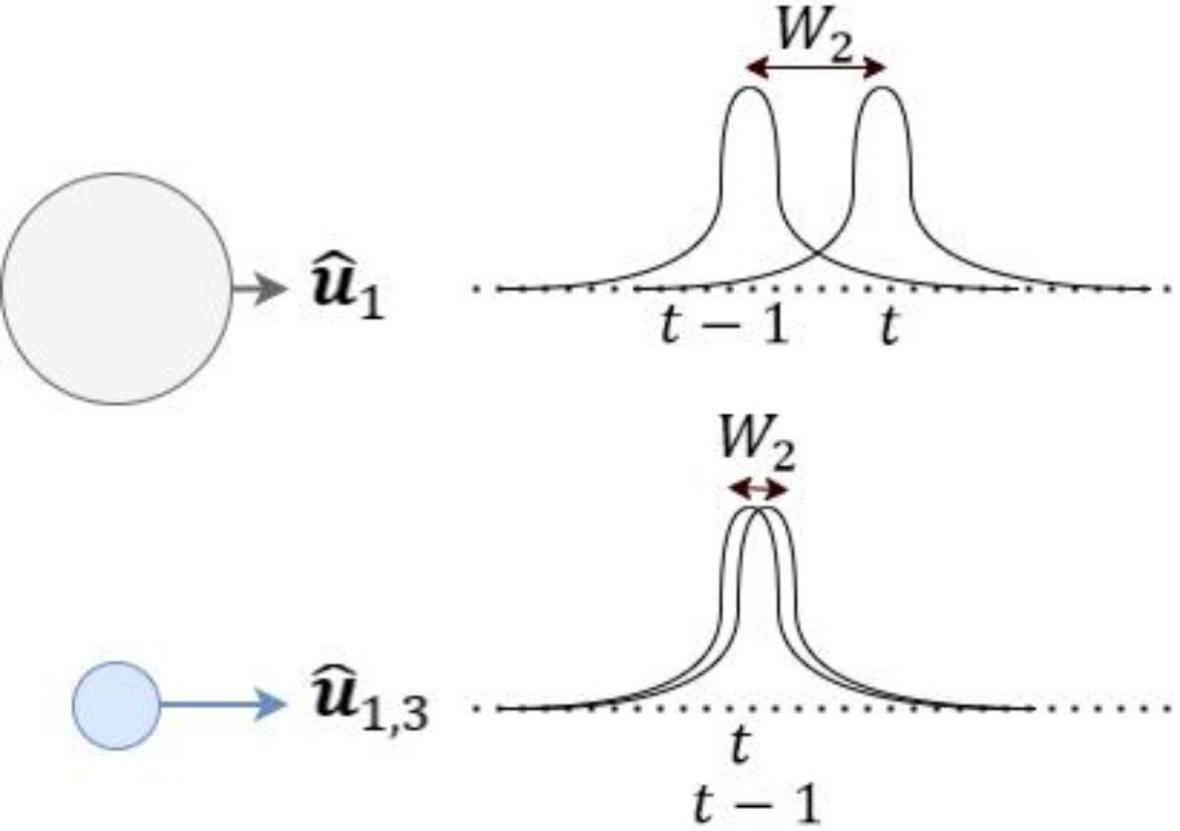
Illustration of the influence of each node on the output distribution, as quantified by the behavior function. For instance, processing $\it  {\widehat{\text{u}}}_{1,\text{t}}$ with the model $\it \text{f}$ results in an output distribution that deviates from the source distribution. Conversely, $\it {\widehat{\text{u}}}_{\text{1,3},\text{t}}$ enables the model to generate a more accurate output, bringing its distribution closer to the source distribution. The distance between these distributions is represented by the Wasserstein distance ${\text{W}}_{2}$.

Following the evaluation of the weighted probability for each node, it is essential to assess its role in aligning the overall output distribution of the model with the source distribution of the training labels (recall Assumptions 1 and 2). To accomplish this, we introduce a *behavior function,* which quantifies the similarity between the distributions of the training labels and the model’s overall outputs factoring in the value of the node under evaluation (see Fig. 3). For classification models, this assessment is conducted for each refined logit ${\widehat{u}}_{i,t}^{*}$. For regression models, the evaluation is performed individually for each logit and its corresponding masks.

The comparison between distributions can be quantified using the Wasserstein distance function ${\text{W}}_{2}$^30^, which measures the similarity between two distributions of two groups of data points, as illustrated in Fig. 3. The behavior function $b$ is thus defined for the logit and, in the case of regression models, for each of its masks as follows,

8 $$b({\widehat{u}}_{t})={\text{W}}_{2}\left({Y}^{s},{\widehat{Y}}_{t-1}\right)-{\text{W}}_{2}\left({Y}^{s},{\widehat{Y}}_{t}\left(g\left({\widehat{u}}_{t}\right)\right)\right)$$

where ${\widehat{u}}_{t}$ can be substituted by the logit or the mask whose behavior needs to be quantified. ${Y}^{s}$ is the vector of the source labels. ${\widehat{Y}}_{t-1}$ is the model’s overall output vector until time $t-1$. ${\widehat{Y}}_{t}\left(g\left({\widehat{u}}_{t}\right)\right)$ is the updated output vector after incorporating the node value ${\widehat{u}}_{t}$, such that

9 $${\widehat{Y}}_{t}\left(g\left({\widehat{u}}_{t}\right)\right)=\left[{\widehat{Y}}_{t-1},g\left({\widehat{u}}_{t}\right)\right]$$

As indicated in (8), the behavior function $b$ comprises of ${\text{W}}_{2}\left({Y}^{s},{\widehat{Y}}_{t-1}\right)$, indicating the previous distance, i.e., the distance between the distribution of the training labels and the outputs of the model up to time $t-1$, in addition to ${\text{W}}_{2}\left({Y}^{s},{\widehat{Y}}_{t}\left(g\left({\widehat{u}}_{t}\right)\right)\right)$ measuring the new (updated) distance, i.e., the distance between the distribution of the training labels and the outputs of the model after considering the node ${\widehat{u}}_{t}$ at time $t$. This allows the behavior function to describe the influence of the node on the output distribution in two cases. (i) Positive impact, when $b({\widehat{u}}_{t})>0$, means that the new distance is smaller than the previous one, indicating that the overall output distribution is closer to the distribution of the training labels after considering the value of this specific node ${\widehat{u}}_{t}$. (ii) Negative impact, when $b({\widehat{u}}_{t})<0$, which suggests that the node negatively impacts the overall behavior, moving the output distribution away from that of the training labels. If $b({\widehat{u}}_{t})=0$, the node has no effect.

After evaluating the behavior of the nodes, we use this information to calculate the prospect certainty for the alternatives i.e. nodes, based on the prospect theory^25^.

#### Prospect certainty

The prospect theory, as described by^25^, quantifies the degree of certainty associated with a decision alternative. Unlike normative models, this descriptive theory, rooted in psychology, focuses on real alternatives rather than ideal choices. According to the prospect theory, individuals evaluate potential losses and gains, not outcomes, using specific heuristics. The general form of the prospect function $\Omega$, incorporating its value ${\Omega }^{b}$ and weighting ${\Omega }^{w}$ functions, is defined as^25,26^,

10 $$\Omega ={\Omega }^{b}(b){\Omega }^{w}({\widehat{\text{Pr}}}^{w})$$

11 $${\Omega }^{b}\left(b\right)=\left\{\begin{array}{c}{b}^{{\varepsilon }_{1}} , b\ge \epsilon \\ -{\gamma }_{b}{(-b)}^{{\varepsilon }_{2}}, b<\epsilon \end{array}\right.$$

12 $${\Omega }^{w}\left({\widehat{\text{Pr}}}^{w}\right)=\text{exp}\left(-\left(-\text{ln}\left({\widehat{\text{Pr}}}^{w}\right)\right){\gamma }_{w}\right)$$

**Fig. 4:**
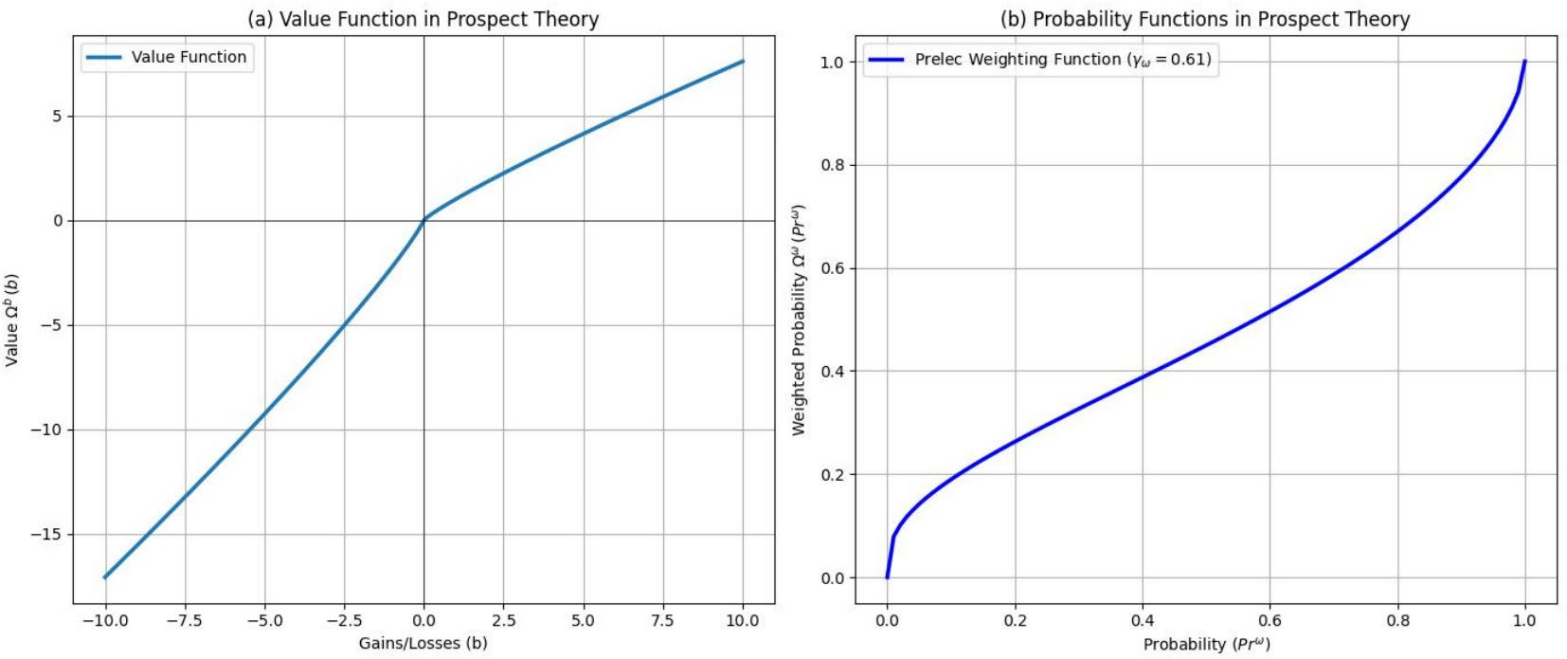
Plots illustrating the behavior of (**a**) the value function ${\Omega }^{\textit{b}}$ is convex and more inclined when $\textit{b}<0$ and concave when $\textit{b}>0$. (**b**) The probability function ${\Omega }^{w}$ is nonlinear and inclines rapidly for values approaching $0$ and $1$. Both functions are components of the prospect index $\Omega$.

The value function ${\Omega }^{b}$ explains how people perceive gains and losses relative to a reference point $\epsilon$, typically the status quo, defined by an expert. This function, characterized by reference dependence, loss aversion, and diminishing sensitivity, is convex for losses i.e. $b<\epsilon$ and concave for gains $b>\epsilon$, as shown in Fig. 4. The Kahneman and Tversky’s experiments^25,26^ yielded specific parameters for ${\varepsilon }_{1}={\varepsilon }_{2}=0.88$, and ${\gamma }_{b}=2.25$, illustrating that losses have a more substantial negative impact than gains have positive effects, as illustrated in Fig. 4.

The probability function ${\Omega }^{w}$ reflects how individuals perceive the probabilities of uncertain outcomes^25,26^. Contrary to traditional expected utility theory^31^, the prospect theory suggests nonlinear probability weighting, exhibiting two key characteristics: overweighting of small probabilities (e.g., overestimating lottery win chances) and underweighting of large probabilities (e.g., underestimating likely losses). The Prelec function (12)^32,33^ captures this curvature as shown in Fig. 4. Setting ${\gamma }_{w}=0.61$ aligns with empirical observations. Notably, ${\Omega }^{b}(0)=0, {\Omega }^{w}(0)=0$, and ${\Omega }^{w}(1)=1$, indicate that for a certain prospect ${\widehat{\text{Pr}}}^{w}=1$, the value and weighting functions coincide, i.e., $\Omega (b,1)={\Omega }^{b}(b)=\Omega (b)$.

**Fig. 5:**
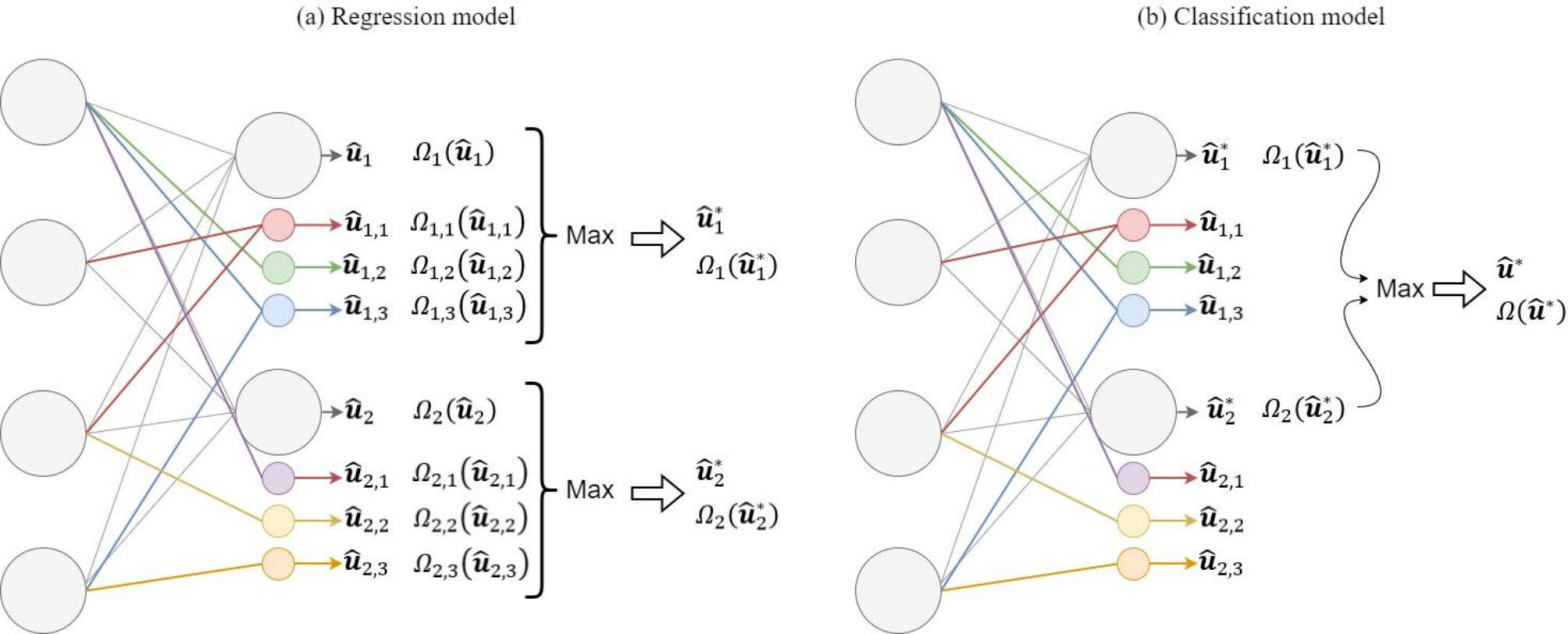
Prospect certainty calculation and refined node selection for (**a**) regression models, where the refined output depends on the prospect certainty of the node and all its masks. (**b**) Classification models, where the refined output is taken considering the refined logits only.

By substituting the behavior function (8) and the weighted probability function (7) into (10), we define the prospect certainty function for data-driven models as follows,

13 $${\Omega }_{t}\left({\widehat{u}}_{t}\right)= {\Omega }^{b}\left(b\left({\widehat{u}}_{t}\right)\right) {\Omega }^{w}\left({\widehat{\text{Pr}}}_{t}^{w}\left({\widehat{u}}_{t}\right)\right)$$

where ${\widehat{u}}_{t}$ denotes the node for which the prospect certainty needs to be evaluated. As illustrated in Fig. 5, the evaluation process varies depending on the model type (i.e., regressor or classifier).

- For regression models, where the logits predict outputs with continuous values, comparing the certainty of individual logits is not meaningful. Therefore, each logit and its associated masks are treated as distinct decision alternatives to measure the certainty of the original logit. This is achieved by substituting the behavior function (8) and the corresponding weighted probability function value (7) for each original logit and its masks separately into (13). Then, to determine the refined value for this logit, ${\widehat{u}}_{i,t}^{*}$, we compare the prospect certainty of the original logit with those of its masks and select the output with the highest prospect certainty value. If multiple nodes exhibit the same certainty, their average value is used. This process is repeated for each logit.
- For classification models, where the models predict a categorical outcome, the refined logits, ${\left\{{\widehat{u}}_{i,t}^{*}\right\}}_{i=1}^{{N}^{L}}$, are considered as decision alternatives for the final output. The value of the certainty function (13) is employed by substituting the value of the behavior function (8) and the corresponding weighted probability function (7) for each refined logit individually. Depending on whether the problem is a multi-class or multi-label classification, the logit or logits with the highest prospect certainty value are selected as final outputs for the model.

The refined logit is then substituted in (2) to yield a more accurate output of the data-driven model,

14 $${\widehat{{\varvec{y}}}}_{t}^{*}=g\left({\widehat{{\varvec{u}}}}_{t}^{*}\right) | {\boldsymbol{\Omega }}_{t}^{*}\left({\widehat{{\varvec{u}}}}_{t}^{*}\right)$$

This formulation implies that the data-driven model provides a refined output or outputs ${\widehat{{\varvec{y}}}}^{*}$ along with their corresponding prospect certainty ${\boldsymbol{\Omega }}^{*}$. Therefore, this approach enables the model to produce results with improved accuracy and explicitly provides their corresponding prospect certainty levels, thereby enhancing the model’s trustworthiness and robustness in a variable environment with distributional changes. The following two algorithms summarize the steps of the proposed approach for regression and classification tasks.

**Algorithm 1:**
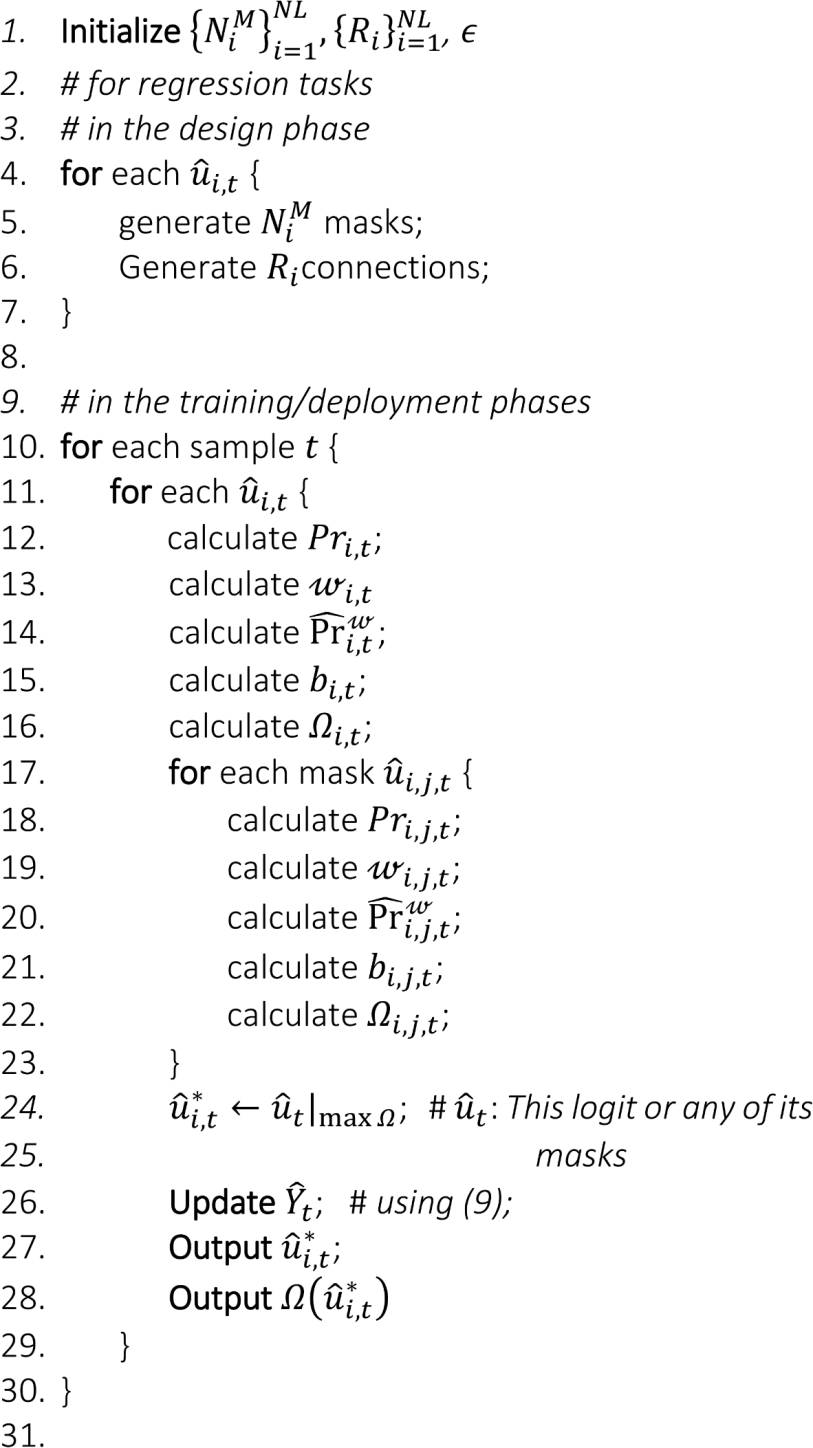
Steps for prospect certainty and output selection for a regression task, showing the functions that are needed to be calculated for each logit and those, required for each of its masks.

**Algorithm 2:**
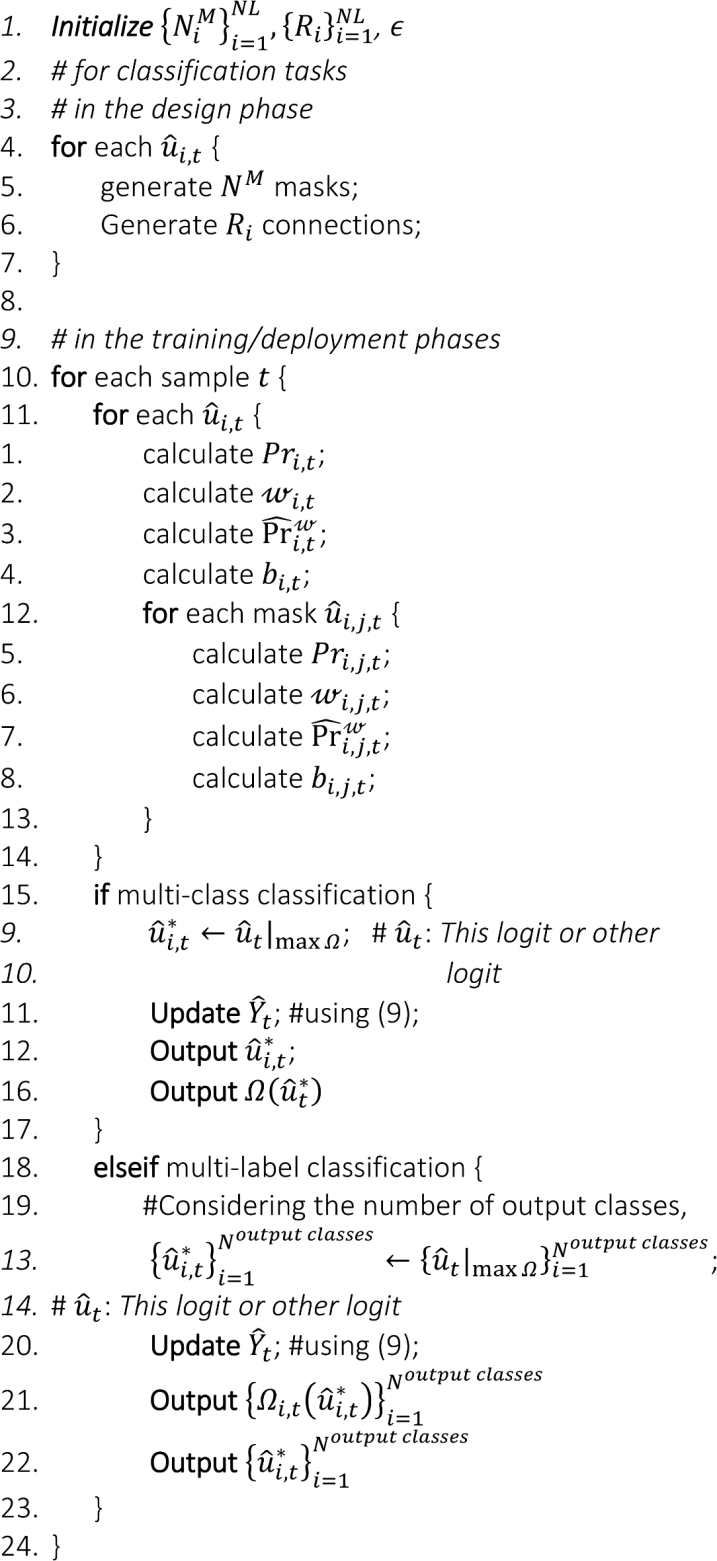
Steps for prospect certainty and output selection for a classification task, illustrating the functions that are required to be calculated for each node, i.e. logit and mask.

### Training procedure

Depending on the prospect theory^25,26^, the application of the proposed prospect certainty method for data-driven models exhibits a linear time complexity of $O({N}^{L})$, where ${N}^{L}$ represents the number of decision alternatives or nodes. Each step in its process, which involves evaluating the value function, applying the probability function, and calculating the certainty index, takes the same computation time per logit. This gives an initial indication, although requires further analyses in subsequent work, that our method is generally fast and efficient, making it suitable for practical applications.

Furthermore, the training process can proceed without any impact on its loss function. Despite its simplicity, our method implicitly enhances the overall accuracy of the model by incorporating prospect certainty in the logit refinement. This improvement is demonstrated in the subsequent experiments.

## Experiments and results discussion

In this section, we evaluate the conformation of the proposed method with Assumptions 1 and 2. We consider two extremely serious situations: a model is confronted with irrelevant samples outside the feature space or the output of the model is trusted. To demonstrate the viability of the proposed method for regression and classification tasks, we conduct two types of experiments using benchmark and real datasets. The accuracy values produced by the model as well as the normalized certainty values serve as the basis for the evaluation criteria. For example, the model is expected to show a high degree of certainty when its output is correct, and a low value when the output is not correct, the input distribution differs from the source, or the input is outside the trained feature space.

### Benchmark experiments for regression

The proposed method is evaluated for regression tasks using a benchmark test case following the model in^34^ to ensure comparability. The implementation uses the TensorFlow $v\text{2.11.0}$. Unless otherwise specified, for the regression-based benchmark experiments, the baseline model of the compared methods is $2 \times Dense(32, ReLU)$ with the following configurations:

- Number of epochs: 700 with early stopping
- Batch size: 32
- Optimizer: Adam with default TensorFlow parameters

The hyperparameters in the prospect certainty method are set as follows:

- Number of masks for each output neuron: ${N}^{M}=3$
- Random connection ratio for each mask: $R\in [0.4, 0.7]$
- Prospect certainty reference point: $\epsilon =0$

The synthetic dataset is generated by sampling the following function ^34^:

15 $$f(x)=x \mathit{sin}x+{h}_{1}x+{h}_{2}$$

where the homoscedastic ${h}_{1}$ and the heteroscedastic ${h}_{2}$ uncertainties are drawn from a Gaussian distribution $\mathcal{N}(0, 0.3)$. In this experiment, we perform two tests with the following data generation:

- In both Test 1 and 2: as a training set, we generate $1000$ samples from $x\in [0, 10]$, while the testing dataset is generated with $200$ i.i.d. samples from $x\in [10, 15]$.
- In Test 2: to mimic a distinct pattern of input uncertainty, high noise data from $\mathcal{N}(-\text{1,1})$ are added to the region $x\in [5, 8]$.

**Fig. 6:**
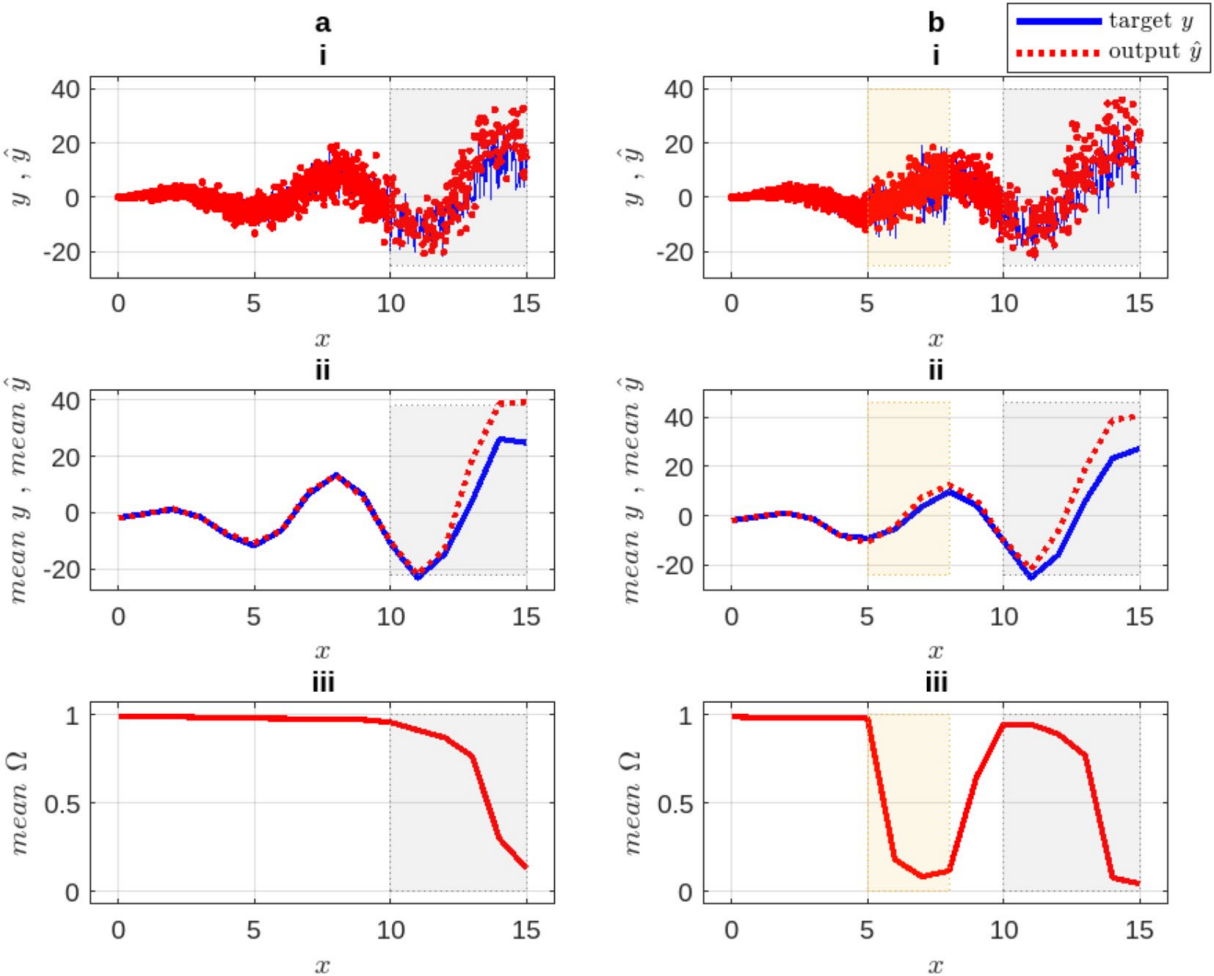
Regression benchmark experiment. (i) Average target and output, (ii) mean curves, and (iii) mean certainty output. Column (**a**) results from the original dataset and (**b**) results after adding the noise.

After running each test five times, the average degrees of certainty and their consistency with the average estimates utilizing our approach, are shown in the Fig. 6.

As seen from Fig. 6, column (a), the normalized certainty index $$\Omega$$ in the period $$x\in [0, 10]$$ is high (almost $$1.0)$$, since the output of the model is as close to the target curve as expected. In column (b), the certainty index in $$x\in [5, 8]$$ is relatively low, since the performance of the model in this range is highly uncertain due to the added noise, despite the output being close to the target. On the other hand, in the testing region $$x\in [10, 15]$$, the model is fitted at first by generating an output that is quite close to the target. But after that, the certainty declines as the difference between the output and the target starts to grow. As a result, the certainty level of the output is adequately reflected by the proposed certainty index $$\Omega$$.

#### The effect of masks and ratios

**Fig. 7:**
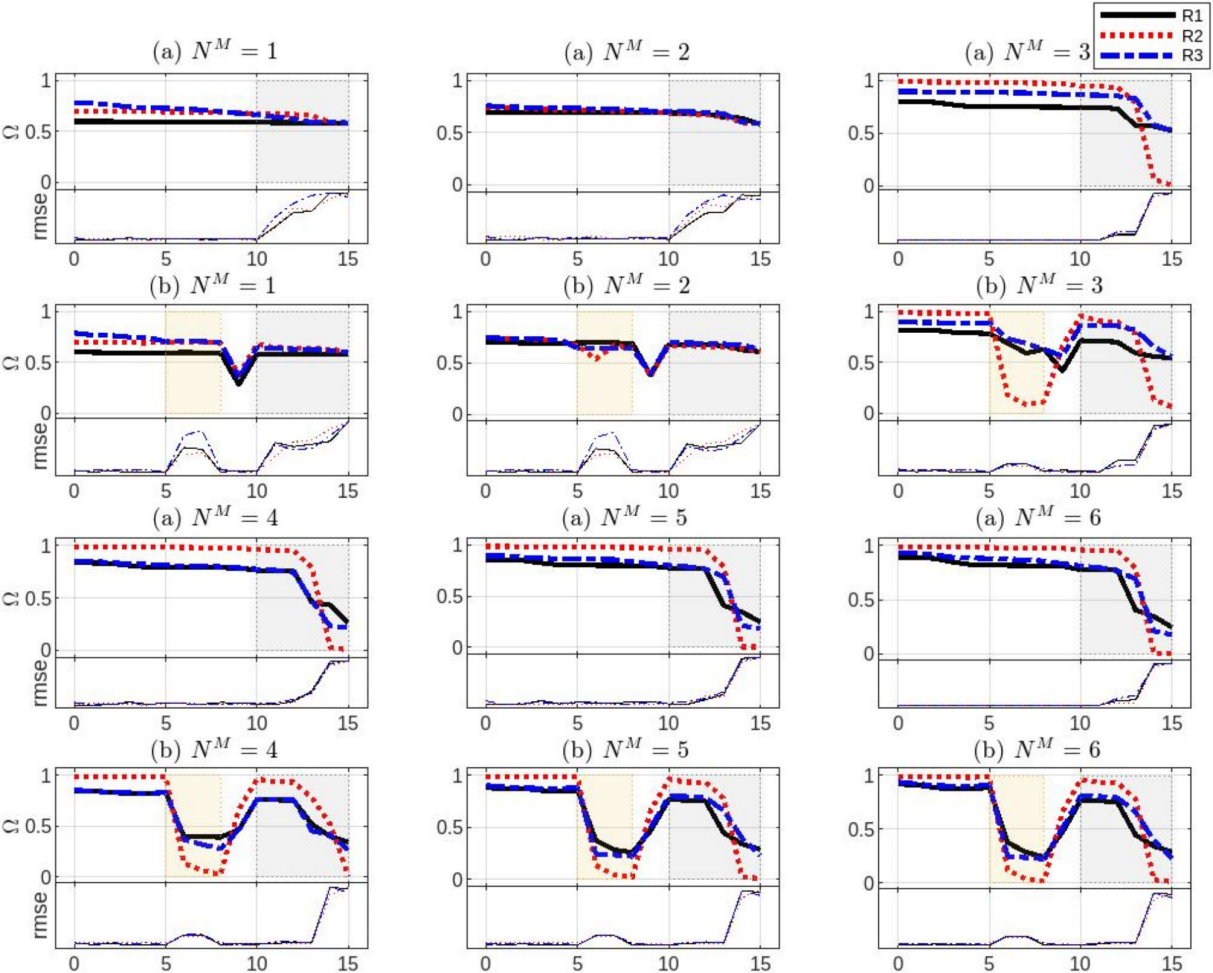
Masks and ratios influence the certainty and error patterns. The rows (**a**) are the results of the original synthetic dataset, while (**b**) after adding the noise.

This experiment aims to evaluate the effects of the number of masks ${N}^{M}$ and the corresponding connection ratios $R$ on the reliability of the certainty index $\Omega$. Six trials are conducted, starting with one mask $({N}^{M}=1)$ in the first trial and increasing the number of masks by one in subsequent trials. In each test, three random connection ratio ranges are used for all the masks, i.e. ${R}_{1}\in [0.2, 0.4], {R}_{2}\in [\text{0.4,0.7}]$, and ${R}_{3}\in [\text{0.7,1.0})$, where $R=1$ denotes a fully connected network. Setting the ratio $R$ within a bounded random range implies that both the indices of the nodes in the subsequent layer and the number of nodes to be connected with are randomly determined for each mask. The aforementioned synthetic datasets are used for this experiment. Each test is performed five times, resulting in the average errors and certainty levels as shown in Fig. 7.

It can be seen from Fig. 7 that the use of only one or two masks proves to be insufficient because $\Omega$ does not accurately reflect either the imprecision or the certainty level in all test regions. This behavior is observed regardless of the random connection ratio of the masks. The influence of $R$ becomes noticeable when three masks are used. Although the pattern of $\Omega$ for ${R}_{1}$ and ${R}_{3}$ shows responses to the error or noise level, the value of $\Omega$ is still not representative. In contrast, the ${R}_{2}$ ratio shows a better response, with $\Omega \cong 1$ in the confident regions and $\Omega \cong 0$ in the uncertain regions. As ${N}^{M}\ge 4$ is used, the certainty $\Omega$ increases, while the impact of $R$ nearly vanishes.

Therefore, ${N}^{M}$ and $R$ as hyperparameters compromise fitting precision and confidence with processing expense. Configuring the masks with low ratios does not provide the necessary amount of information, while higher ratios enable the delivery of more redundant and correlated information, resulting in a more similar or even identical output for each mask.

As a result of this experiment, it is found that utilizing random connection ratios with moderate values allows the masks to be less deterministic and more resistant to overfitting while compensating for the need for a larger number of masks. In the following experiment, we show the model behavior in processing the same synthetic benchmark dataset.

#### Regression results with and without prospect certainty

**Fig. 8:**
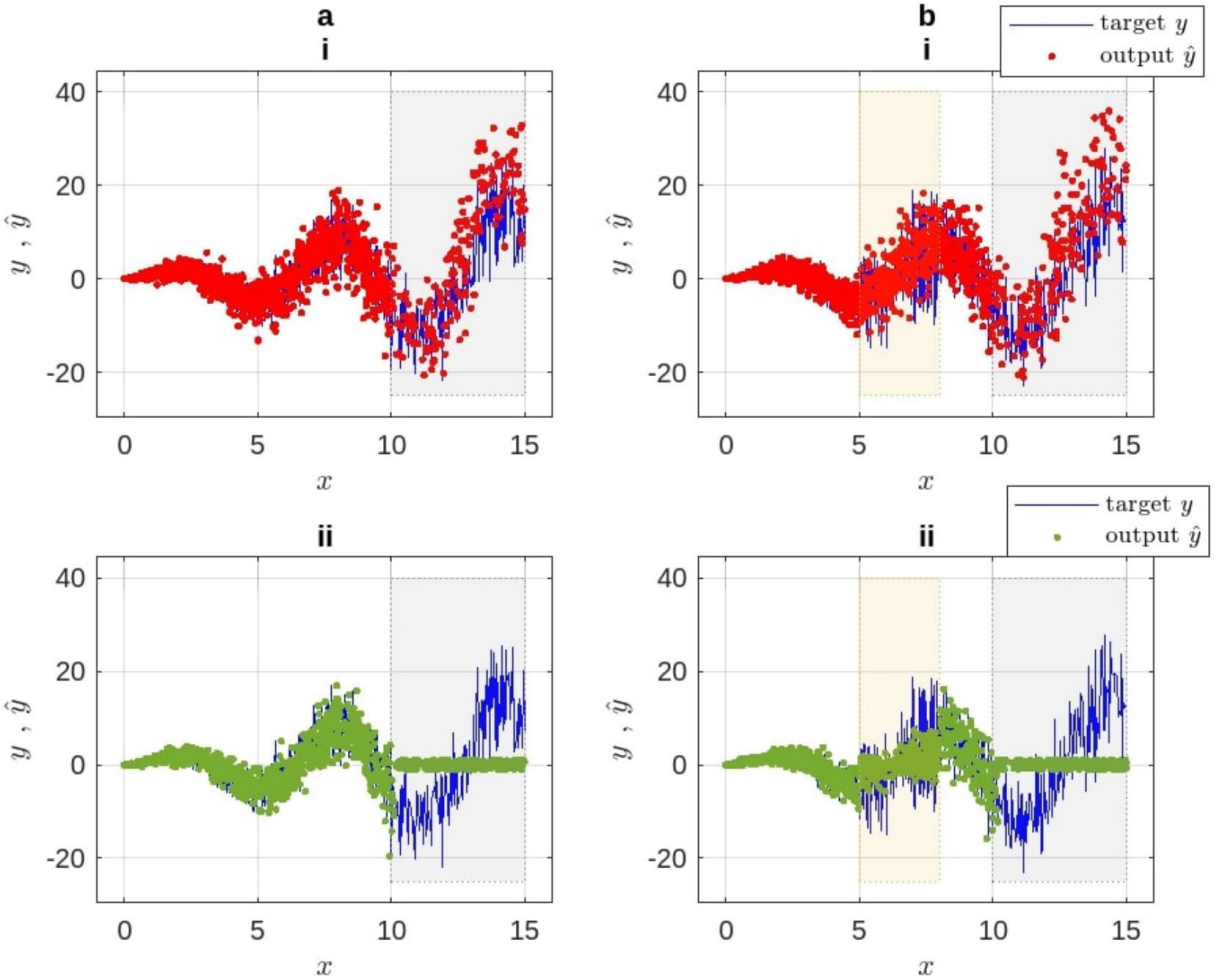
Synthetic benchmark for the regression model, an output comparison between (i) the original model with our prospect certainty, and (ii) without it. (**a**) synthetic dataset, (**b**) after adding the noise.

Figure 8 shows a comparison between the behavior of the original model and that after applying the proposed method.

This experiment confirms that our proposed method implicitly enhances the model’s accuracy. This improvement is due to the added features of less deterministic masks and the prospect certainty evaluation. In the following experiment, we compare the behavior of the model employing our method with it using state-of-the-art techniques.

#### Comparison studies

The proposed approach is compared with; dropout as an approximate Bayesian inference method that relies on sampling^35^, ensemble learning^34^ which uses multiple models, and evidential learning^14,15^ which is based on evidence acquisition. The setup of the compared methods is as follows:

- Dropouts: baseline model with random dropout 25%^35^.
- Ensembles: 5 replicates of the baseline model with random initialization^34^.
- Evidential: with regularization coefficient $\lambda =0.01$^15^.

All methods were run five times on the same synthetic datasets as above. The root mean square error (RMSE) is shown in Table 1. It is worth noting that RMSE considers the deviation between the output and the target even in cases where the certainty index is low, which makes this comparison unfair to our method.

**Table 1:** Results of the regression experiment using state-of-the-art methods and the proposed approach.

Method: Baseline +	Root mean square error $\downarrow$				
	Without noise		With noise		
	Training (i.i.d.)$x \in [0, 10]$	Testing $x \in [10, 15]$	Training: Training (i.i.d.)$x \in \{[0, 6],[\text{8,10}]\}$	Training: Training (noise)$x\in [6, 8]$	Testing $x \in [10, 15]$
Ensembles	00.07	03.68	00.08	01.78	04.02
Dropout	00.10	03.01	00.12	02.30	04.11
Evidential	**00.03**	01.07	**00.04**	01.49	03.82
Our method	00.05	**00.21**	00.07	**00.72**	**00.95**

Significant values are given in bold.

From Table 1, it can be generally observed that the divergence between the training and testing results by our method is smaller compared with the other methods. This is because of our method’s capability to consider the distributional changes of input data. Moreover, it is seen that the evidential method reaches the best accuracy for the i.i.d. regions, followed by our method. However, in the truly uncertain regions, i.e., the testing and noisy regions, our method shows a significant improvement in accuracy with the lowest error. This increases the credibility of the model since learning with the prospect certainty index enables the model to provide a higher prediction accuracy even in cases with considerable uncertainty.

### Benchmark experiments for classification

We conduct a benchmark classification to test not only the overall accuracy but also the behavior of the certainty index for individual cases. In this experiment, training is done with the six classes of Cifar-10^36^ (i.e. bird, dog, automobile, ship, horse, and airplane). ImageNet^37^ and the COCO^38^ datasets are used only for testing to simulate the distributional changes. To enable an accurate evaluation, the common categories from the testing datasets are pooled and given the same index as in the training dataset.

The baseline model used for this experiment is ResNet-9, whose architecture is described in^39^. Each model is set with 120 epochs, a batch size of 64, and trained with Adam in the default setting with no differential privacy. If the validation loss does not improve in three epochs, a constant learning rate is set with a reduction of half. Batch normalization is applied with the default parameters. The Optuna package^40^ with the tree-structured Parzen estimator^41^ is used to determine the values of the other hyperparameters.

To evaluate the prospect certainty for new single patterns, we enter a single image at a time and measure the output of the system along with its normalized prospect certainty. Table 2 shows a sample of the results.

**Table 2:**
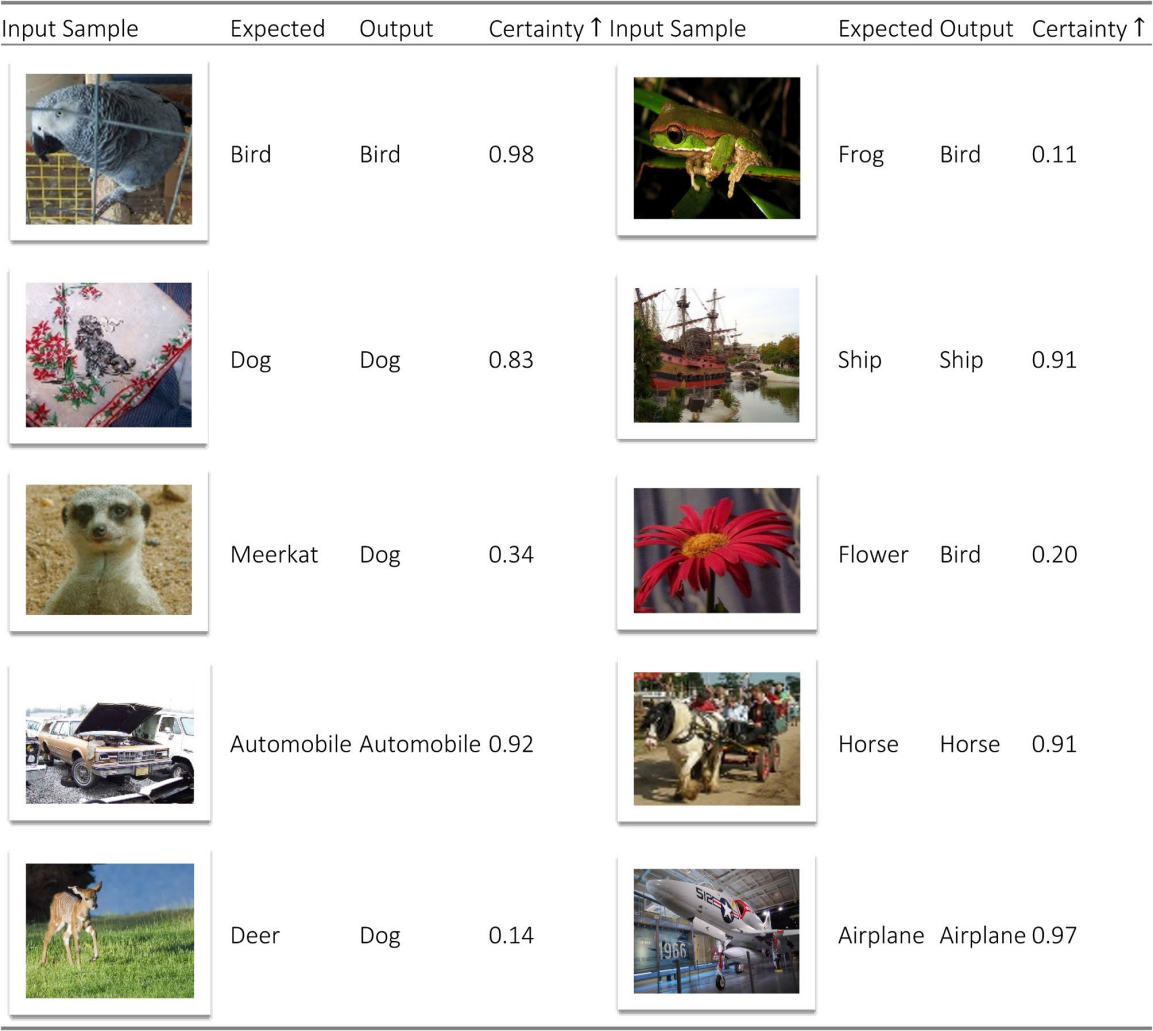
Sampled normalized prospect certainty index responses in the benchmark classification task.

Table 2 compares the model output vs predictions of selected input images, showing the correlation between the prospect certainty index and the accuracy. Since only six specific classes are used to train the model, it produces an output using one of these classes for each input case. Nevertheless, it can be seen from Table 2 that our approach proves its reliability by displaying a low prospect certainty $\Omega$ each time the model produces incorrect output. This provides the level of *uncertainty* of the model.

To evaluate the impact of the certainty index on the error, we suggest a new error measurement scheme: if the model is uncertain about its output, the error of that output should not be considered in the overall error measurement. This is implemented by computing the categorical cross-entropy error index of the output node $i$ as follows,

16 $${L}_{i}=\left\{\begin{array}{c}0, \Omega <0.5\\ -log\left({\mathbb{E}}\left({\widehat{y}}_{i,t}|{u}_{t}\right)\right), otherwise\end{array}\right.$$

Table 3 shows the overall accuracy of the model after implementing the proposed method with and without using the refined error measurement (16).

**Table 3:** Mean cross-entropy error values for the classification with and without the refined loss (16) using three benchmark datasets.

Method	Mean cross-entropy error $\downarrow$		
	CIFAR-10	ImageNet	COCO
Baseline + proposed method without error refinement	$0.10$	$1.11$	$1.90$
Baseline + proposed method with error refinement	${2.0e}^{-3}$	${4.4e}^{-2}$	${6.1e}^{-2}$

From Table 3, it can be seen that the value of the certainty index has a noticeable impact on the overall results. This is a clear result that using an explicit certainty index for the output improves the reliability of the model in real-world applications.

#### Comparison studies for the classification experiment

In this benchmark classification experiment, the mean cross-entropy (MCE) loss measure is used for comparison between our method and the existing methods. All methods were run five times. Table 4 shows the mean error from each method on each dataset.

**Table 4:** Comparison of results for the classification by the state-of-the-art and our method using three benchmark datasets.

Method	Mean cross-entropy error $\downarrow$		
	CIFAR-10	ImageNet	COCO
ResNet-9	0.29	12.89	19.29
ResNet-9 + Ensembles	0.15	08.22	11.68
ResNet-9 + Dropout	0.14	07.56	14.45
ResNet-9 + Evidential	0.13	07.02	07.99
ResNet-9 + Our method	**0.10**	**01.11**	**01.90**

Significant values are given in bold.

It is seen from Table 4 that our proposed solution outperforms the state-of-the-art methods with a significant increase in accuracy. This is attributed to the logit refinement through the masking principle in addition to the prospect certainty which accounts for the influence of each decision alternative i.e. logit in the output distribution. However, it is important to acknowledge the potential limitations, such as a relative increase of the computational complexity and resource requirements in comparison with the baseline as illustrated in the experiment with the real dataset.

It should be noted that the certainty measure shown in Table 4 is not comparative, due to the lack of a technique for calculating the certainty for each test sample in the compared methods. Hence, the certainty index output of our method is not considered here. Therefore, the result here does not accurately reflect the behavior of our approach. To make a comparative assessment of predictive certainty, we test the models on five additional classes outside of those used in the training phase, by ensuring that they do not share labels with the trained classes. This test should be expected to have a high error rate, as all test samples are anticipated to be miscategorized. As a result, the level of certainty of the models for each test sample should be quite low. By our approach, we estimate the certainty using the prospect certainty function (13). For comparison, we use the entropy function as described in^42^ as an uncertainty index for all compared methods, since they considered the entropy to have a positive correlation with the uncertainty.

**Fig. 9:**
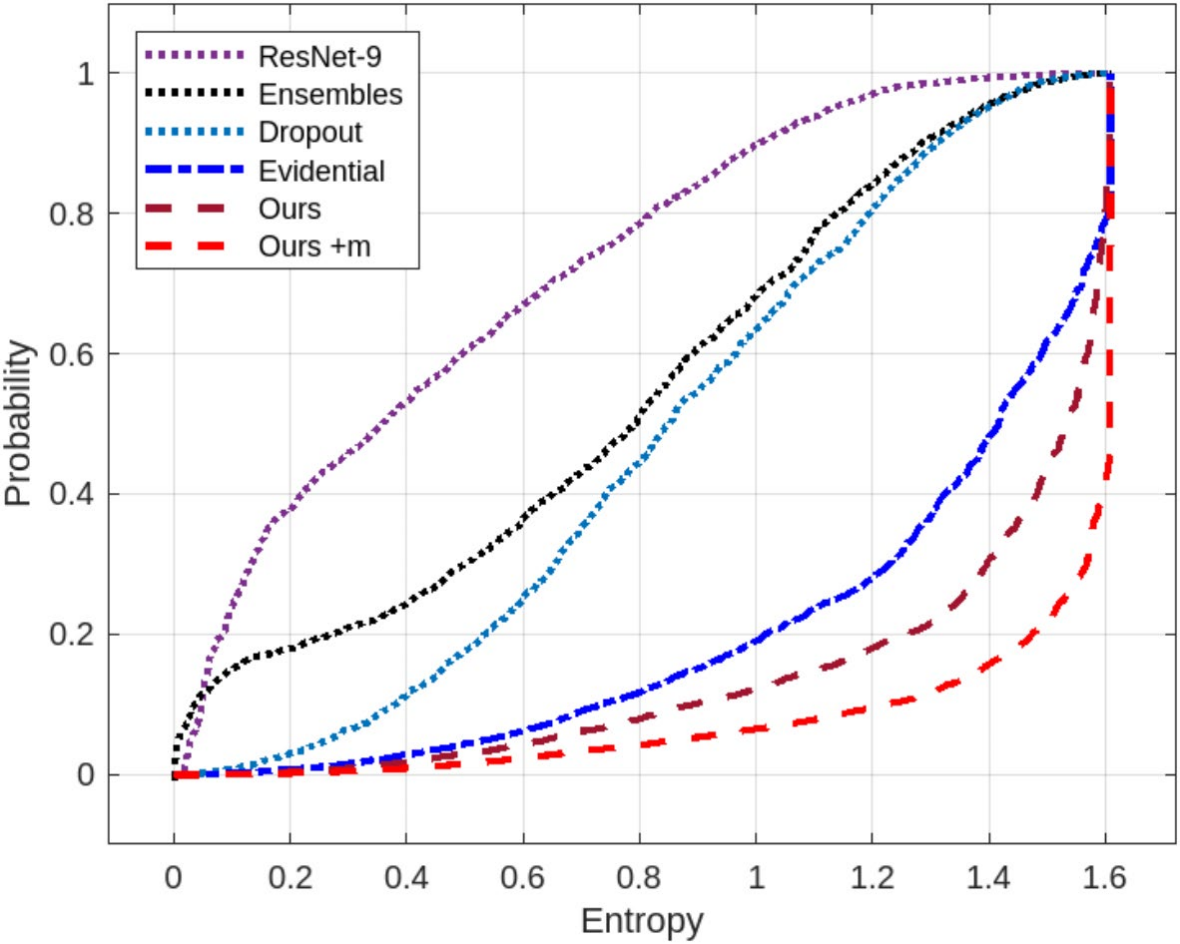
ECDFs comparison for the benchmark classification. The red dashed plot (of our method) is shown near the bottom right corner, indicating that the classification entropy is low, in correlation with the classification accuracy.

Figure 9 shows the empirical CDFs over the range of possible entropies of the testing classes from CIFAR-10, ImageNet, and COCO for the compared methods. It can be seen that the curve obtained by our method is on the far right, indicating that it has the lowest prediction certainty compared to the state-of-the-art methods. This is because the test samples, in this experiment, do not belong to the feature space of the training dataset. Therefore, the model does not provide a correct output consequently, i.e. its certainty should be low, which is clearly reflected by our method.

### Experiment with a real-world dataset

To replicate a variable environment with distribution changes, pedestrian detection and tracking to estimate their intention on roads is considered. This application is significant since it involves human beings, which is a very valuable agent. Moreover, numerous applications, such as autonomous vehicles or real-time surveillance systems, sensitively and crucially depend on intention detection. Furthermore, the fact that the variations of agents and objects in the environment cannot be enumerated, i.e. its environment is highly variable in the distribution that can occur at any time during the deployment period.

Pedestrian intention estimation involves three main tasks: a classification and two regression tasks for tracking and estimating the future position, which are briefly outlined below:

- Pedestrian detection and classification: The goal of this task is to determine whether the objects in an image are pedestrians or not. For this purpose, CNN-M-2048 is adopted from^43^ and fine-tuned for the binary classification. It consists of five convolutional layers and three fully connected layers.
- Pedestrian tracking: This task involves locating the detected pedestrians by bounding boxes and then tracking them. For this purpose, an LSTM model is adopted from^44^ and added after the convolutional layers of CNN-M-2048 in parallel with the fully connected layer. Together with the four attribute vectors of the bounding boxes, this LSTM model outputs the confidence and the association degree. To explain the input and output settings of this pipeline, let ${f}_{i}$ be the input frame, where $i$ is an integer number. Then, the input of the neural network at time *t* is ${{\varvec{x}}}_{t}={f}_{i}$. The output of the detection, classification, and tracking tasks is ${\widehat{\mathcal{Y}}}_{t}=[{{\varvec{c}}}_{t}, {\mathcal{x}}_{t},{\mathcal{y}}_{t},{{\varvec{w}}}_{t},{{\varvec{h}}}_{t}]$, where ${\varvec{c}}$ is the class vector of the detected objects, $\mathcal{x},$ and $\mathcal{y}$ are the center position vectors of the bounding boxes ${{\varvec{b}}{\varvec{b}}}_{t}$. ${\varvec{h}}$ and ${\varvec{w}}$ are their height and width vectors, respectively.
- Future position prediction: To estimate the future locations in terms of expected bounding box coordinates, only pedestrians that are reliably identified (i.e., confident > 0.9) are processed. The LSTM model from^44^ is also adapted to achieve this goal; it accepts the frame ${f}_{i}$ along with the output of the previous tasks ${\widehat{{\mathcal{Y}}}_{t}}$ as inputs, s.t. ${{\varvec{x}}}_{t}=\left\{{f}_{i},{\widehat{\mathcal{Y}}}_{t}|confident>0.9\right\}$, and generates the bounding box attributes for two times ahead i.e. ${{\varvec{b}}{\varvec{b}}}_{t+2}$. It is worth noting that the movement difference from one frame to the next is essentially negligible. Hence, in this task, a 5-frame skip is regularly performed for the input. For example, the inputs in the interval ${t}_{0}$ to ${t}_{2}$ are ${x}_{{t}_{0}}={f}_{0}$, ${x}_{{t}_{1}}={f}_{5}$, ${x}_{{t}_{2}}={f}_{10}$. Consequently, the output of the predictor estimates the future bounding box after 10 frames. i.e. ${\widehat{{\varvec{y}}}}_{t}=[{\mathcal{x}}_{i+10},{\mathcal{y}}_{i+10},{{\varvec{w}}}_{i+10},{{\varvec{h}}}_{i+10}]$. For instance, the output for ${{\varvec{x}}}_{{t}_{0}}={f}_{0}$ is the bounding box vector ${\widehat{{\varvec{y}}}}_{{t}_{0}}=[{\mathcal{x}}_{10},{\mathcal{y}}_{10},{{\varvec{w}}}_{10},{{\varvec{h}}}_{10}]$ which represents the projected future locations of pedestrians after 10 frames. The second input, ${{\varvec{x}}}_{{t}_{1}}={f}_{5}$, then leads to the second output, ${\widehat{{\varvec{y}}}}_{{t}_{1}}=[{\mathcal{x}}_{15},{\mathcal{y}}_{15},{{\varvec{w}}}_{15},{{\varvec{h}}}_{15}]$, and so on.

In this experiment, the PIE dataset^45^ is chosen to train the models, whereas the JAAD dataset^46^, the PANDA dataset^47^, and the Waymo Perception dataset^48^ are utilized for testing. All experiments and modifications are performed using TensorFlow v2.11.0. Both CNN-M-2048 and LSTM models are set as,

- Number of epochs: 120 with early stopping set to 3 epochs.
- Batch size: 64,
- Optimizer: Adam in the default setting.
- Constant learning rate reduction: 0.5.
- Batch normalization: activated.
- Optuna^40^ with a tree-structured Parzen estimator^41^ is used to determine the values of the other hyperparameters. More details can be found in^44^.

#### Classification results

For this task, the following hyperparameters are used in the prospect certainty method:

- $${N}^{M}=3$$
- $$R\in [\text{0.4,0.7}]$$
- Prospect certainty reference point: $\epsilon =0$.

Table 5 shows the outcomes of the binary classification estimates (i.e. pedestrian or not pedestrian) with different approaches. The mean average precision $mAP$ is used to calculate the results for each method over all the tests.

**Table 5:** Comparison between the state-of-the-art and out method considering $\text{mAP}$ and average inference speed in milliseconds for the classification.

Method	$mAP \uparrow$				Average inference speed ($ms$)$\downarrow$
	PIE	JAAD	PANDA	Waymo perception	
Baseline	*84.16*	*46.26*	*33.01*	*34.78*	*00.74*
Baseline + Ensembles	83.54	59.71	39.67	47.99	03.12
Baseline + Dropout	84.91	62.22	40.26	49.90	02.99
Baseline + Evidential	85.79	60.79	49.05	48.37	01.20
Baseline + Our method	**87.05**	**82.12**	**79.22**	**81.47**	**00.81**

Significant values are given in bold.

The results show that our method provides an improved accuracy at a tiny higher processing cost compared to the baseline. This is due to the additional processes and neurons (i.e. masks). However, our method demonstrates significant improvements in accuracy and processing speed over the state-of-the-art methods. This is because our method is not based on sampling. In addition, the added processes in our method have comparatively low complexity. Therefore, it can be considered a viable approach for real-time applications where the input data distribution constantly changes.

#### Regression results

Turning to the regression task for intention detection, the following hyperparameters are used in our method:

- $${N}^{M}=3$$
- $$R\in [\text{0.4,0.7}]$$
- Prospect certainty reference point: $\epsilon =0$.

The results of the bounding box predictions after 10 frames for each of the compared approaches are shown in Table 6. The results are obtained by taking the average intersection over union (AIoU) with comparing the values of the estimated position with the ground truth of the associated dataset. It is important to note that the accuracy of the detection and classification task directly affects the prediction results.

**Table 6:** Comparison between the state-of-the-art and our method using $\text{AIoU}$ and average inference speed in milliseconds for the regression.

Method	$AIoU \uparrow$				Average inference speed ($ms$)$\downarrow$
	PIE	JAAD	PANDA	Waymo perception	
Baseline	*41.11*	*19.14*	*14.26*	*17.43*	*00.25*
Baseline + Ensembles	44.17	28.10	27.04	27.97	02.09
Baseline + Dropout	45.32	27.12	23.44	25.74	01.72
Baseline + Evidential	45.89	30.33	28.05	27.87	00.86
Baseline + Our method	**49.25**	**41.82**	**39.38**	**41.09**	**00.52**

Significant values are given in bold.

Considering the trade-off between the accuracy of the model and the processing speed, it can be seen that, in comparison to the baseline, although our method improves the accuracy, it requires a little more processing time since it contains more computations and neurons (i.e. masks). At the same time, the performance of the used model is considerably improved with our method in comparison to the state-of-the-art.

It is important to note that the results presented in Tables 5 and 6 were computed independently to eliminate any dependencies and ensure the integrity of the analysis. Additionally, the overall low accuracy results shown in Tables 4, 5, and 6 across all the compared methods are attributed to the use of the baseline models. These models serve as fundamental benchmarks and can be significantly improved by employing more sophisticated deep learning architectures and optimization techniques, which is beyond the scope of this study. The baseline models in this work are intentionally selected due to their simplicity, which facilitates a clearer comparison of the methods and effectively demonstrates the scope of this study.

In summary, the results of the conducted experiments show that the proposed method can highly improve the accuracy of neural network models with low processing time. This demonstrates its capability for real-world online applications.

## Conclusions

This paper addresses the problem of quantifying the certainty of outputs in data-driven models with input distributional changes or new samples beyond the trained feature space. Given the inherent uncertainties within both the model and the data drawn from unknown distributions, it becomes essential to measure the certainty of each decision the model produces, especially since data-driven models deterministically generate outputs for every given input. Such determinism can degrade the output accuracy when the model encounters inputs from unfamiliar distributions. This paper introduces a novel method for explicitly quantifying the certainty of a model’s output, referred to as *prospect certainty*. First, we introduce the concept of *logit masking* which generates new masks for selected output logits. These masks provide alternative decisions for each logit, leading to a probabilistic layer to the model and mitigating its deterministic nature. Second, we define a *weighted probability function* to assess the dispersion of these alternatives and gain initial insights into their certainty. Third, we evaluate the effect of each decision alternative on the model’s output distribution by a *behavior function* that measures the distance between the output distribution, influenced by a potential decision alternative, and the distribution of the training data. Using these measures, we quantify the *prospect certainty* for each decision alternative, finally refining the model’s final output.

For regression models, the final output is refined by comparing the prospect certainty indices of the logits and their corresponding masks and selecting the node with the highest certainty index. In classification models, the refined logits serve as decision alternatives for the prospect certainty evaluation, with the logit or logits having the highest certainty values chosen as the final outputs, tailored to the classification type. As a result, the proposed approach enhances the accuracy of the model’s outputs and provides a quantified measure of output certainty, thereby improving the robustness and reliability of data-driven models in real-world settings with varying input distributions. Our experimental results demonstrate the effectiveness of the proposed method by showing a positive relationship between the certainty index and the model’s accuracy. This underscores the importance of considering both the model’s behavior and the overall output pattern when selecting outputs and determining their certainty levels. The sources of uncertainty that models may encounter often overlap, making explicit quantification vital to the applicability of the model in real-world applications.

However, the computational complexity, scalability, generalizability, and resource requirements of the proposed method should be further studied to ensure its stability, usability, and efficiency of the prospect certainty in diverse settings. In addition, a specified level of the certainty index could be considered as a constraint for the process of training the model. Moreover, logit masking and the corresponding ratios can be expanded for use in different layers of the model in addition to the output logits.

## Supplementary Information

Supplementary Information 1.

Supplementary Information 2.

## Data Availability

Publicly available datasets: The datasets used to validate the proposed method are listed below with the corresponding references. However, some of the datasets require registration for the user to download them. The CIFAR-10 dataset is available at https://www.cs.toronto.edu/~kriz/cifar.html. Described in: Alex Krizhevsky, "Learning Multiple Layers of Features from Tiny Images," in 2009. [Online]. Available: https://api.semanticscholar.org/CorpusID:18268744. The ImageNet dataset is available at: https://www.image-net.org/download.php. Described in: O. Russakovsky et al., "ImageNet Large Scale Visual Recognition Challenge," Int J Comput Vis, vol. 115, no. 3, pp. 211–252, 2015, doi: 10.1007/s11263-015-0816-y. The COCO dataset is available at: https://cocodataset.org/#download. Described in: T.-Y. Lin et al., "Microsoft COCO: Common Objects in Context," in Lecture Notes in Computer Science, Computer Vision – ECCV 2014, D. Fleet, T. Pajdla, B. Schiele, and T. Tuytelaars, Eds., Cham: Springer International Publishing, 2014, pp. 740–755. The PIE dataset is available at: https://data.nvision2.eecs.yorku.ca/PIE_dataset/. Described in: A. Rasouli, I. Kotseruba, T. Kunic, and J. Tsotsos, "PIE: A Large-Scale Dataset and Models for Pedestrian Intention Estimation and Trajectory Prediction," in 2019 IEEE/CVF International Conference on Computer Vision (ICCV), 2019, pp. 6261–6270. The JAAD dataset is available at: https://data.nvision2.eecs.yorku.ca/JAAD_dataset/. Described in: A. Rasouli, I. Kotseruba, and J. K. Tsotsos, "Are They Going to Cross? A Benchmark Dataset and Baseline for Pedestrian Crosswalk Behavior," in 2017 IEEE International Conference on Computer Vision Workshops (ICCVW), 2017, pp. 206–213. The PANDA dataset is available at: https://gigavision.cn/track/track/?nav=Tracking. Described in: X. Wang et al., "PANDA: A Gigapixel-level Human-centric Video Dataset," 2020. The Waymo dataset is available at: https://waymo.com/open/data/perception/. Described in: P. Sun et al., "Scalability in Perception for Autonomous Driving: Waymo Open Dataset," in Proceedings of the IEEE/CVF Conference on Computer Vision and Pattern Recognition (CVPR), 2020.
