# Supplementary material for: Prospect certainty for data-driven models
Source: Sci Rep. 2025 Mar 10;15:8278. doi: 10.1038/s41598-025-89679-6 (PMC11893788; doi:10.1038/s41598-025-89679-6)
Supplement: Supplementary file 1 — Supplementary Information 1. [file 41598_2025_89679_MOESM1_ESM.docx]

Type of the Paper (Research)

Title *Prospect certainty for data-driven models*

Qais Yousef ^1,^* & Pu Li ^2,^*

1 ORCID: [0000-0003-0239-9150].

2 ORCID: [0000-0001-6481-9961].

1,2 Group of Process Optimization, Institute for Automation and Systems Engineering, Technische Universität Ilmenau, P.O. Box 100565, 98684 Ilmenau, Germany

* Correspondence: {qais.yousef, pu.li}@tu-ilmenau.de

**Synthetic Dataset**

| **Original Data** | | **Noisy Data** | |
| --- | --- | --- | --- |
| **Input** | **Output** | **Input** | **Output** |
| 0.0179 | -0.0031 | 0.0148 | 0.0025 |
| 0.0202 | -0.0099 | 0.0398 | 0.0020 |
| 0.0227 | -0.0024 | 0.0427 | 0.0261 |
| 0.0359 | -0.0039 | 0.0429 | -0.0227 |
| 0.0392 | -0.0145 | 0.0547 | 0.0090 |
| 0.0421 | 0.0112 | 0.0684 | -0.0464 |
| 0.0518 | 0.0101 | 0.1266 | -0.0119 |
| 0.0566 | 0.0216 | 0.1298 | -0.0052 |
| 0.0592 | 0.0114 | 0.1360 | 0.0790 |
| 0.0610 | 0.0195 | 0.1408 | -0.0474 |
| 0.0747 | -0.0171 | 0.1464 | -0.0635 |
| 0.0904 | 0.0393 | 0.1520 | 0.0274 |
| 0.0932 | -0.1066 | 0.1538 | -0.0433 |
| 0.1027 | 0.1044 | 0.1657 | 0.0365 |
| 0.1057 | 0.0501 | 0.1684 | 0.1662 |
| 0.1089 | -0.0013 | 0.1723 | 0.2347 |
| 0.1178 | 0.0177 | 0.1842 | 0.0117 |
| 0.1228 | 0.0203 | 0.1923 | -0.1578 |
| 0.1255 | 0.0618 | 0.1952 | 0.0575 |
| 0.1311 | 0.0325 | 0.1965 | -0.0389 |
| 0.1360 | 0.0890 | 0.2025 | -0.0248 |
| 0.1484 | 0.0334 | 0.2107 | -0.0825 |
| 0.1669 | 0.0242 | 0.2521 | -0.1013 |
| 0.1676 | 0.1322 | 0.2979 | 0.0905 |
| 0.1739 | -0.0257 | 0.3405 | 0.2042 |
| 0.2030 | 0.1267 | 0.3473 | 0.1036 |
| 0.2044 | -0.0497 | 0.3614 | -0.1366 |
| 0.2241 | 0.0019 | 0.3656 | 0.1492 |
| 0.2261 | -0.0823 | 0.3757 | 0.3999 |
| 0.2269 | 0.0135 | 0.3859 | 0.4941 |
| 0.2278 | -0.0256 | 0.4043 | -0.0348 |
| 0.2282 | 0.2850 | 0.4045 | 0.3179 |
| 0.2345 | 0.0626 | 0.4049 | -0.0553 |
| 0.2470 | 0.0659 | 0.4241 | 0.0336 |
| 0.2474 | 0.0666 | 0.4277 | 0.1423 |
| 0.2477 | 0.1772 | 0.4297 | 0.1701 |
| 0.2482 | 0.0838 | 0.4462 | 0.2825 |
| 0.2562 | 0.0818 | 0.4587 | 0.1156 |
| 0.2599 | 0.0051 | 0.5007 | 0.5904 |
| 0.2600 | 0.0383 | 0.5107 | 0.1541 |
| 0.2747 | 0.0478 | 0.5212 | 0.1985 |
| 0.3155 | 0.1398 | 0.5222 | 0.2314 |
| 0.3158 | 0.2591 | 0.5311 | 0.0563 |
| 0.3300 | 0.1142 | 0.5393 | 0.5095 |
| 0.3399 | 0.0953 | 0.5396 | 0.2415 |
| 0.3474 | 0.0656 | 0.5466 | 0.2359 |
| 0.3539 | 0.1287 | 0.5653 | -0.1003 |
| 0.3700 | 0.0072 | 0.5951 | 0.0793 |
| 0.3740 | 0.2490 | 0.6043 | -0.1588 |
| 0.3781 | 0.2858 | 0.6079 | 0.3195 |
| 0.3839 | 0.1104 | 0.6191 | 0.6165 |
| 0.3987 | 0.1317 | 0.6212 | 0.6839 |
| 0.4124 | 0.2538 | 0.6259 | 0.2516 |
| 0.4127 | 0.1827 | 0.6260 | 0.5489 |
| 0.4167 | 0.3572 | 0.6342 | 11.846 |
| 0.4225 | 0.2233 | 0.6352 | -0.1310 |
| 0.4276 | 0.3054 | 0.6353 | 0.6684 |
| 0.4340 | 0.1278 | 0.6368 | 0.4047 |
| 0.4411 | -0.0882 | 0.6421 | 0.1746 |
| 0.4437 | 0.0168 | 0.6422 | 0.6414 |
| 0.4458 | 0.7523 | 0.6492 | 0.2454 |
| 0.4612 | 0.3779 | 0.6581 | 0.9479 |
| 0.4770 | 0.3088 | 0.6881 | -0.0974 |
| 0.4795 | 0.0434 | 0.6952 | 0.6761 |
| 0.4799 | 0.1978 | 0.7058 | 0.1758 |
| 0.4983 | 0.3416 | 0.7071 | 0.4267 |
| 0.4990 | 0.2170 | 0.7340 | 0.8836 |
| 0.5166 | 0.1142 | 0.7379 | 0.6091 |
| 0.5363 | 0.1266 | 0.7439 | 0.5204 |
| 0.5364 | 0.2259 | 0.7442 | 0.5754 |
| 0.5456 | 0.2825 | 0.7458 | 14.404 |
| 0.5936 | 0.6693 | 0.7738 | 10.058 |
| 0.6012 | 0.0654 | 0.7759 | 0.1979 |
| 0.6096 | 0.6866 | 0.7834 | 0.5255 |
| 0.6222 | 0.2490 | 0.7839 | 0.7626 |
| 0.6234 | 0.5688 | 0.7879 | 0.5215 |
| 0.6270 | 0.3901 | 0.7896 | 0.6534 |
| 0.6388 | 0.1721 | 0.8387 | 0.6015 |
| 0.6456 | 0.5796 | 0.8470 | 0.5903 |
| 0.6626 | 0.1694 | 0.8515 | 0.3716 |
| 0.6881 | 0.6226 | 0.8519 | 0.5055 |
| 0.7002 | 0.7856 | 0.8549 | 0.3470 |
| 0.7053 | -0.3431 | 0.8688 | 0.6768 |
| 0.7081 | 0.3667 | 0.9151 | 10.848 |
| 0.7147 | 0.1125 | 0.9316 | 0.2662 |
| 0.7173 | 0.1735 | 0.9497 | 11.360 |
| 0.7213 | 0.4170 | 0.9555 | 14.863 |
| 0.7220 | 0.5182 | 0.9602 | 0.1374 |
| 0.7252 | 0.0016 | 0.9639 | 0.7737 |
| 0.7312 | 0.4311 | 0.9756 | 11.640 |
| 0.7500 | 0.4398 | 0.9760 | 13.480 |
| 0.7657 | 0.7677 | 0.9833 | 0.9005 |
| 0.7685 | 0.7686 | 10.076 | 0.3774 |
| 0.7691 | 0.2748 | 10.169 | 0.9675 |
| 0.7974 | 0.3725 | 10.300 | 14.796 |
| 0.7982 | 0.5996 | 10.416 | 0.9306 |
| 0.8238 | 0.6413 | 10.484 | 0.4870 |
| 0.8359 | 13.193 | 10.512 | 12.144 |
| 0.8562 | 0.7154 | 10.784 | 13.389 |
| 0.8814 | 0.5348 | 11.037 | 0.2124 |
| 0.9039 | 0.6497 | 11.196 | 10.014 |
| 0.9080 | 10.929 | 11.411 | 0.8039 |
| 0.9222 | 13.032 | 11.466 | 0.8541 |
| 0.9242 | 0.4717 | 11.575 | 11.619 |
| 0.9327 | 0.7512 | 11.635 | 0.2907 |
| 0.9557 | 0.9825 | 11.646 | 13.907 |
| 0.9707 | 11.641 | 11.716 | 15.641 |
| 0.9721 | 0.3811 | 11.770 | 10.473 |
| 0.9840 | 0.7498 | 11.788 | 11.267 |
| 0.9911 | 10.551 | 11.934 | 15.740 |
| 0.9939 | 12.278 | 12.022 | 0.8878 |
| 10.004 | 0.8713 | 12.142 | 0.6908 |
| 10.128 | 0.9697 | 12.393 | 0.9697 |
| 10.167 | 10.453 | 12.492 | 10.187 |
| 10.212 | 12.163 | 12.561 | 0.5606 |
| 10.301 | 0.4198 | 12.645 | 16.495 |
| 10.335 | 0.6145 | 12.727 | 13.829 |
| 10.796 | 0.8705 | 12.863 | 0.4902 |
| 10.886 | 12.512 | 12.895 | 0.9134 |
| 10.932 | 0.8848 | 13.026 | 13.919 |
| 11.025 | 13.842 | 13.134 | 15.566 |
| 11.080 | 10.822 | 13.161 | 15.401 |
| 11.316 | 15.509 | 13.191 | 18.269 |
| 11.623 | 11.316 | 13.282 | 16.251 |
| 11.692 | 10.182 | 13.346 | 18.763 |
| 11.733 | 11.838 | 13.420 | 11.165 |
| 11.850 | 10.610 | 13.493 | 0.9622 |
| 12.022 | 20.241 | 13.521 | 10.209 |
| 12.058 | 16.965 | 13.524 | 0.6926 |
| 12.096 | 11.779 | 13.789 | 17.919 |
| 12.102 | 18.520 | 14.022 | 16.923 |
| 12.338 | 0.0986 | 14.115 | 13.872 |
| 12.492 | 14.212 | 14.191 | 19.558 |
| 12.585 | 15.499 | 14.296 | 14.254 |
| 12.825 | 11.826 | 14.404 | 0.6378 |
| 12.837 | 0.8540 | 14.509 | 0.6674 |
| 12.971 | 18.045 | 14.576 | 18.108 |
| 13.048 | 0.7111 | 14.596 | 16.491 |
| 13.266 | 0.8422 | 14.627 | 12.768 |
| 13.474 | 19.219 | 14.919 | 13.232 |
| 13.554 | 0.6544 | 15.054 | 19.307 |
| 13.683 | 13.392 | 15.067 | 15.773 |
| 13.761 | 0.7392 | 15.104 | 0.8779 |
| 13.943 | 20.560 | 15.365 | 16.491 |
| 13.987 | 21.728 | 15.418 | 24.296 |
| 14.228 | 15.671 | 15.463 | 20.398 |
| 14.238 | 15.476 | 15.493 | 0.3865 |
| 14.261 | 17.092 | 15.531 | 14.757 |
| 14.417 | 23.245 | 15.539 | 24.120 |
| 14.436 | 15.033 | 15.696 | 19.533 |
| 14.637 | 0.9883 | 15.721 | 0.1703 |
| 14.716 | 0.5617 | 15.744 | 21.045 |
| 14.993 | 11.627 | 15.749 | 0.6737 |
| 15.000 | 11.188 | 15.783 | 18.951 |
| 15.017 | 0.9272 | 15.796 | 18.753 |
| 15.059 | 0.8594 | 15.888 | 0.8298 |
| 15.073 | 17.750 | 15.942 | 28.712 |
| 15.211 | 19.602 | 15.964 | 0.7957 |
| 15.325 | 16.612 | 16.081 | 18.559 |
| 15.348 | 17.885 | 16.195 | 11.890 |
| 15.432 | 0.9078 | 16.477 | 18.569 |
| 15.462 | 14.804 | 16.483 | 16.306 |
| 15.576 | 0.5406 | 16.807 | 33.665 |
| 15.649 | 15.230 | 16.904 | 22.207 |
| 15.810 | 0.8706 | 17.047 | 13.093 |
| 15.866 | 10.095 | 17.069 | 15.935 |
| 15.936 | 13.747 | 17.154 | 11.852 |
| 15.949 | 25.095 | 17.351 | 12.876 |
| 16.102 | 22.912 | 17.409 | 17.829 |
| 16.198 | 0.9422 | 17.450 | 18.598 |
| 16.306 | 0.7208 | 17.491 | 24.844 |
| 16.327 | 24.170 | 17.607 | 0.6859 |
| 16.352 | 21.410 | 17.670 | 12.579 |
| 16.564 | 0.5192 | 17.688 | 15.568 |
| 16.626 | 23.126 | 17.832 | 0.7176 |
| 16.746 | 34.331 | 18.046 | 19.929 |
| 16.942 | 14.385 | 18.317 | 11.107 |
| 16.992 | 23.087 | 18.336 | 20.119 |
| 17.009 | 16.335 | 18.521 | 24.376 |
| 17.228 | 23.411 | 18.531 | 25.701 |
| 17.310 | 15.024 | 18.564 | 0.8219 |
| 17.328 | 12.375 | 18.772 | 13.149 |
| 17.382 | 0.9142 | 18.776 | 0.9922 |
| 17.506 | 22.340 | 18.779 | 15.021 |
| 17.528 | 12.802 | 18.919 | 16.282 |
| 17.804 | 13.115 | 19.091 | 19.319 |
| 17.920 | 0.8431 | 19.190 | 18.438 |
| 18.031 | 11.320 | 19.258 | 0.1577 |
| 18.091 | 18.951 | 19.295 | 21.159 |
| 18.240 | 15.843 | 19.382 | 0.2094 |
| 18.253 | 21.149 | 19.478 | 18.725 |
| 18.328 | 28.079 | 19.718 | 17.720 |
| 18.391 | 14.418 | 19.790 | 16.574 |
| 18.397 | 36.736 | 19.897 | 25.266 |
| 18.561 | 18.760 | 19.900 | 0.3261 |
| 18.624 | 18.715 | 19.924 | 0.5849 |
| 18.635 | 27.524 | 19.924 | 31.090 |
| 18.776 | 18.405 | 19.946 | 33.691 |
| 18.947 | -0.1464 | 19.993 | 13.455 |
| 18.976 | 32.735 | 20.004 | 32.437 |
| 19.010 | 16.657 | 20.027 | 21.975 |
| 19.032 | 25.548 | 20.194 | 15.014 |
| 19.032 | 0.5739 | 20.405 | 24.594 |
| 19.109 | 0.9049 | 20.412 | 22.910 |
| 19.147 | 26.872 | 20.596 | 10.140 |
| 19.172 | 22.010 | 20.611 | 11.425 |
| 19.361 | 14.596 | 20.619 | 28.421 |
| 19.440 | -0.5357 | 20.668 | 24.545 |
| 19.466 | 11.351 | 20.706 | 19.668 |
| 19.561 | 18.202 | 20.715 | 0.6021 |
| 19.665 | 16.945 | 20.729 | 14.868 |
| 19.849 | 0.9945 | 20.797 | 13.333 |
| 19.861 | 11.035 | 20.834 | 0.6449 |
| 20.025 | 12.551 | 20.841 | 20.100 |
| 20.035 | 18.863 | 21.011 | 11.299 |
| 20.068 | 0.4265 | 21.124 | -0.3577 |
| 20.141 | 0.3322 | 21.133 | 22.778 |
| 20.214 | 25.621 | 21.167 | 17.388 |
| 20.250 | 28.528 | 21.236 | 22.022 |
| 20.276 | 12.582 | 21.254 | 16.394 |
| 20.470 | 13.287 | 21.267 | 19.029 |
| 20.729 | 21.952 | 21.309 | 16.713 |
| 20.744 | 0.9006 | 21.314 | 29.094 |
| 20.785 | 13.477 | 21.526 | -0.0233 |
| 20.806 | 11.898 | 21.540 | 23.681 |
| 20.866 | 0.9680 | 21.574 | 18.870 |
| 21.007 | 14.747 | 21.796 | 0.8617 |
| 21.023 | 15.711 | 21.810 | 17.477 |
| 21.076 | 0.6954 | 21.853 | 14.932 |
| 21.139 | 15.239 | 21.921 | 19.713 |
| 21.159 | 30.144 | 21.965 | 36.817 |
| 21.187 | 12.816 | 22.023 | 18.473 |
| 21.279 | 12.982 | 22.027 | 10.353 |
| 21.364 | 16.237 | 22.090 | 21.589 |
| 21.486 | 25.416 | 22.232 | 0.8752 |
| 21.550 | 32.047 | 22.286 | 34.427 |
| 21.644 | 15.441 | 22.373 | 13.078 |
| 21.784 | 15.059 | 22.548 | 28.398 |
| 21.844 | 19.489 | 22.579 | 22.770 |
| 21.890 | 18.244 | 22.724 | 0.2059 |
| 21.948 | 0.0390 | 22.778 | 14.335 |
| 22.021 | 0.4047 | 23.021 | -10.355 |
| 22.181 | 0.4074 | 23.040 | 0.3450 |
| 22.313 | 25.472 | 23.372 | -0.6873 |
| 22.816 | 12.351 | 23.392 | -0.2338 |
| 22.894 | 23.363 | 23.456 | 0.7214 |
| 22.982 | 20.283 | 23.463 | 21.055 |
| 23.009 | 32.318 | 23.627 | 18.261 |
| 23.288 | 0.9805 | 23.760 | 20.221 |
| 23.294 | 12.685 | 24.167 | 0.9282 |
| 23.338 | 17.572 | 24.471 | 14.080 |
| 23.415 | 13.199 | 24.912 | 22.059 |
| 23.505 | 21.982 | 24.932 | 26.873 |
| 23.517 | 21.564 | 25.273 | 0.4547 |
| 23.527 | 13.642 | 25.288 | 23.165 |
| 23.545 | 0.8798 | 25.331 | 27.289 |
| 23.604 | 39.663 | 25.553 | 0.3618 |
| 23.694 | 0.6760 | 25.610 | 11.335 |
| 23.697 | 0.2461 | 25.679 | 29.219 |
| 23.757 | 25.362 | 25.704 | 0.8779 |
| 23.788 | 23.431 | 25.795 | 0.8287 |
| 23.881 | 0.1935 | 25.927 | 11.142 |
| 23.912 | 0.4117 | 25.951 | 0.1435 |
| 23.998 | 21.593 | 26.099 | 21.980 |
| 24.011 | 14.250 | 26.170 | 0.0474 |
| 24.070 | 17.463 | 26.354 | 19.543 |
| 24.240 | 15.022 | 26.415 | 0.7905 |
| 24.288 | 11.250 | 26.762 | -0.2067 |
| 24.299 | 12.283 | 26.821 | 36.514 |
| 24.306 | 13.263 | 26.832 | -0.6918 |
| 24.487 | 15.557 | 26.841 | 14.471 |
| 24.548 | 27.076 | 27.198 | 0.5381 |
| 24.630 | 14.301 | 27.295 | 13.383 |
| 24.801 | 18.359 | 27.353 | 17.736 |
| 24.839 | 0.6259 | 27.619 | 27.863 |
| 24.886 | 20.452 | 27.756 | 19.551 |
| 25.048 | 35.383 | 27.759 | 22.776 |
| 25.161 | 0.5962 | 27.770 | 0.9865 |
| 25.233 | 23.535 | 27.774 | 12.360 |
| 25.247 | 25.161 | 27.821 | 32.396 |
| 25.318 | 34.042 | 27.828 | 13.143 |
| 25.394 | 11.900 | 27.861 | 26.156 |
| 25.410 | 25.922 | 28.061 | 18.566 |
| 25.482 | 11.123 | 28.192 | 13.471 |
| 25.628 | 13.365 | 28.317 | 0.2716 |
| 25.662 | 14.664 | 28.361 | 0.4673 |
| 25.726 | 13.908 | 28.381 | 17.706 |
| 25.735 | -0.8527 | 28.450 | 15.005 |
| 25.782 | 14.495 | 28.459 | 0.3075 |
| 25.875 | 12.863 | 28.618 | 29.966 |
| 25.880 | 0.8967 | 28.794 | -0.9359 |
| 26.075 | 19.824 | 28.798 | 0.6029 |
| 26.252 | 14.675 | 28.967 | 0.3512 |
| 26.416 | 0.7039 | 29.035 | 32.982 |
| 26.667 | 0.9689 | 29.061 | 13.727 |
| 26.823 | 29.901 | 29.151 | 14.978 |
| 26.929 | 19.333 | 29.189 | -0.0856 |
| 26.994 | 11.257 | 29.376 | 0.7893 |
| 27.089 | 0.9677 | 29.393 | 13.522 |
| 27.101 | 0.8526 | 29.464 | 0.5629 |
| 27.308 | 0.2441 | 29.495 | -13.780 |
| 27.313 | 13.517 | 29.716 | 0.0827 |
| 27.324 | 11.851 | 29.753 | 0.1659 |
| 27.340 | 15.918 | 29.952 | -0.8783 |
| 27.467 | -0.0396 | 30.034 | 0.9411 |
| 27.470 | 0.7640 | 30.040 | 20.805 |
| 27.774 | 13.442 | 30.093 | -0.8049 |
| 27.989 | 0.7440 | 30.142 | 0.3910 |
| 28.153 | 12.484 | 30.216 | 0.9430 |
| 28.292 | 0.1334 | 30.280 | 0.0381 |
| 28.310 | 18.835 | 30.587 | -0.2705 |
| 28.320 | 19.940 | 30.615 | 26.995 |
| 28.564 | 17.745 | 30.717 | -13.826 |
| 28.717 | 22.899 | 30.770 | 14.174 |
| 28.907 | 10.092 | 30.817 | -0.7500 |
| 29.054 | -0.3105 | 30.852 | -0.7386 |
| 29.233 | 0.9354 | 30.853 | -14.995 |
| 29.321 | 0.8246 | 30.863 | -0.5001 |
| 29.404 | 29.580 | 30.959 | 0.4630 |
| 29.494 | 0.7599 | 31.112 | 0.9426 |
| 29.506 | 0.6574 | 31.131 | -0.2029 |
| 29.638 | 0.8540 | 31.212 | 0.4634 |
| 29.813 | 29.158 | 31.252 | -11.836 |
| 29.893 | -0.0994 | 31.268 | -0.6765 |
| 29.900 | 19.617 | 31.282 | 12.986 |
| 30.033 | 13.675 | 31.405 | -0.6426 |
| 30.120 | 0.3666 | 31.438 | 0.0274 |
| 30.243 | 31.159 | 31.727 | -19.664 |
| 30.363 | -0.1166 | 32.137 | 0.4343 |
| 30.418 | -0.9876 | 32.183 | 15.741 |
| 30.466 | -11.027 | 32.643 | -0.5250 |
| 30.846 | -0.2942 | 32.685 | -0.4383 |
| 30.849 | 12.921 | 32.774 | 0.1432 |
| 30.952 | 0.5632 | 32.879 | -10.605 |
| 31.028 | 0.6892 | 32.971 | -13.466 |
| 31.097 | 0.9710 | 33.023 | 0.2083 |
| 31.136 | 10.352 | 33.061 | 0.6642 |
| 31.140 | 0.1692 | 33.090 | -18.483 |
| 31.567 | 13.871 | 33.099 | -0.8216 |
| 31.588 | -0.0437 | 33.187 | 10.247 |
| 31.688 | 0.8623 | 33.238 | -10.438 |
| 31.753 | 0.8950 | 33.320 | -24.190 |
| 31.846 | 0.5927 | 33.391 | 28.416 |
| 31.940 | -10.228 | 33.410 | 21.599 |
| 31.954 | -28.460 | 33.420 | -12.733 |
| 32.023 | -29.643 | 33.616 | -0.9914 |
| 32.023 | -14.183 | 33.628 | -0.5003 |
| 32.078 | -16.476 | 33.636 | 0.0734 |
| 32.172 | -0.6335 | 33.666 | -27.106 |
| 32.316 | 18.168 | 33.707 | 0.4308 |
| 32.519 | 11.086 | 33.837 | 15.435 |
| 32.524 | -37.060 | 33.949 | -31.568 |
| 32.545 | -0.4784 | 33.993 | -31.958 |
| 32.589 | 0.2040 | 34.341 | 0.7918 |
| 32.614 | -0.2392 | 34.379 | -0.8333 |
| 32.670 | 12.291 | 34.499 | -46.579 |
| 32.882 | -0.1583 | 34.511 | -14.571 |
| 32.897 | -18.586 | 34.688 | -10.246 |
| 32.974 | 24.084 | 34.742 | -0.6278 |
| 32.989 | 15.852 | 35.070 | -16.362 |
| 32.995 | -12.105 | 35.078 | -29.792 |
| 33.011 | 0.2073 | 35.137 | 0.1971 |
| 33.075 | 0.8081 | 35.284 | -0.8581 |
| 33.090 | -23.605 | 35.456 | -39.438 |
| 33.265 | 0.0079 | 35.567 | -34.912 |
| 33.321 | -15.099 | 35.641 | 37.084 |
| 33.390 | 10.414 | 36.058 | 10.986 |
| 33.517 | -0.8276 | 36.094 | -28.512 |
| 33.643 | -34.862 | 36.145 | -25.941 |
| 33.650 | -19.434 | 36.152 | -0.3299 |
| 33.742 | -26.709 | 36.272 | -14.338 |
| 33.776 | -0.3394 | 36.427 | -23.689 |
| 33.920 | 20.743 | 36.448 | -33.914 |
| 33.971 | -16.520 | 36.476 | -56.204 |
| 34.014 | -19.188 | 36.620 | -13.256 |
| 34.064 | -0.1281 | 36.653 | -11.851 |
| 34.122 | -0.7638 | 36.664 | -35.966 |
| 34.195 | 0.1777 | 36.699 | -19.446 |
| 34.210 | -10.705 | 36.751 | -15.325 |
| 34.327 | -19.199 | 36.777 | -29.507 |
| 34.455 | -24.991 | 36.812 | -37.134 |
| 34.623 | -17.030 | 36.831 | -0.1963 |
| 34.687 | -0.8684 | 36.939 | 22.823 |
| 34.706 | 0.8904 | 37.049 | -12.888 |
| 34.891 | -47.380 | 37.069 | -28.601 |
| 34.894 | -12.439 | 37.168 | 0.5062 |
| 34.906 | -18.556 | 37.271 | -11.444 |
| 34.909 | -15.139 | 37.361 | -28.442 |
| 34.967 | -15.547 | 37.398 | 0.3481 |
| 35.144 | -24.034 | 37.717 | -41.648 |
| 35.195 | -26.500 | 37.777 | -26.229 |
| 35.259 | 0.6176 | 37.842 | -0.7429 |
| 35.336 | -21.745 | 37.981 | -0.7077 |
| 35.840 | -23.669 | 38.011 | -60.504 |
| 35.853 | -32.381 | 38.256 | -31.969 |
| 35.884 | -23.653 | 38.703 | -25.113 |
| 36.036 | -0.0538 | 38.779 | -17.117 |
| 36.200 | 0.6381 | 38.803 | -47.338 |
| 36.273 | -10.857 | 38.813 | -36.101 |
| 36.318 | -26.374 | 38.993 | -64.550 |
| 36.390 | -15.886 | 39.204 | -42.352 |
| 36.403 | 0.7478 | 39.211 | -49.308 |
| 36.408 | 0.5829 | 39.259 | -58.494 |
| 36.673 | -18.605 | 39.371 | -33.253 |
| 36.754 | 13.043 | 39.445 | -36.521 |
| 36.757 | -15.290 | 39.487 | -32.350 |
| 36.787 | -29.222 | 39.500 | -25.245 |
| 36.968 | -51.991 | 39.507 | -29.442 |
| 37.192 | -13.985 | 39.509 | -36.942 |
| 37.230 | -49.091 | 39.553 | -17.628 |
| 37.368 | -26.638 | 39.592 | -45.534 |
| 37.522 | -10.442 | 39.690 | -36.418 |
| 37.643 | -12.966 | 40.011 | -14.965 |
| 37.734 | 11.628 | 40.163 | -42.003 |
| 37.773 | -0.7124 | 40.171 | -62.429 |
| 37.958 | -14.119 | 40.258 | -0.9441 |
| 38.023 | -29.964 | 40.379 | -30.880 |
| 38.162 | -22.062 | 40.390 | -44.305 |
| 38.357 | -58.937 | 40.733 | -36.844 |
| 38.371 | -19.873 | 40.866 | -0.9098 |
| 38.753 | -0.6624 | 40.873 | -19.935 |
| 38.785 | -44.885 | 40.910 | -46.581 |
| 38.834 | -12.922 | 40.941 | -14.428 |
| 38.988 | -25.026 | 40.962 | -27.529 |
| 39.244 | -0.6898 | 40.991 | -25.955 |
| 39.255 | -40.083 | 41.098 | -19.417 |
| 39.398 | -32.683 | 41.136 | -46.336 |
| 39.496 | -25.406 | 41.236 | -23.236 |
| 39.506 | -0.6031 | 41.242 | -16.633 |
| 39.549 | -45.333 | 41.374 | -44.783 |
| 39.622 | -60.216 | 41.493 | -45.920 |
| 39.862 | -14.274 | 41.522 | -22.380 |
| 40.109 | -11.010 | 41.608 | -23.613 |
| 40.179 | -25.404 | 41.625 | -32.644 |
| 40.268 | -19.135 | 41.730 | -43.929 |
| 40.459 | -66.888 | 41.961 | -14.266 |
| 40.479 | -49.192 | 42.180 | -52.801 |
| 40.481 | -48.442 | 42.185 | -31.869 |
| 40.499 | -41.002 | 42.384 | -48.872 |
| 40.571 | -0.3915 | 42.581 | -77.165 |
| 40.947 | -40.692 | 42.623 | 0.3676 |
| 40.972 | -21.201 | 42.698 | -30.340 |
| 40.996 | -34.772 | 42.765 | -20.171 |
| 41.037 | -42.162 | 42.969 | -53.134 |
| 41.122 | -31.187 | 42.981 | -45.940 |
| 41.176 | -31.269 | 43.044 | -22.358 |
| 41.227 | -16.159 | 43.259 | -46.176 |
| 41.431 | -48.085 | 43.294 | -26.855 |
| 41.445 | -23.824 | 43.383 | -18.382 |
| 42.116 | -71.210 | 43.426 | -33.300 |
| 42.425 | -51.720 | 43.497 | -41.590 |
| 42.584 | -24.694 | 43.806 | -34.471 |
| 42.585 | -68.208 | 43.975 | -58.764 |
| 42.622 | -43.483 | 44.036 | -61.719 |
| 42.671 | -48.391 | 44.081 | -29.777 |
| 43.054 | -55.968 | 44.103 | -54.022 |
| 43.176 | -16.704 | 44.121 | -55.791 |
| 43.452 | 0.2345 | 44.161 | -29.331 |
| 43.508 | -58.374 | 44.203 | -40.888 |
| 43.583 | -41.998 | 44.299 | -40.879 |
| 43.710 | -30.498 | 44.384 | -32.589 |
| 43.720 | -73.828 | 44.517 | -48.061 |
| 43.820 | -19.297 | 44.681 | -55.887 |
| 43.836 | -66.172 | 44.726 | -24.367 |
| 43.946 | -61.668 | 44.898 | -42.048 |
| 43.948 | -21.968 | 44.907 | -67.730 |
| 43.977 | -23.581 | 44.928 | -32.744 |
| 44.146 | -44.260 | 45.003 | -0.7099 |
| 44.315 | -46.744 | 45.028 | -19.771 |
| 44.319 | -49.709 | 45.075 | -30.633 |
| 44.477 | -22.035 | 45.366 | -37.231 |
| 44.484 | -38.535 | 45.676 | -33.861 |
| 44.600 | -48.363 | 46.191 | -47.031 |
| 44.842 | -52.215 | 46.219 | -33.069 |
| 44.946 | -37.393 | 46.353 | -62.237 |
| 45.017 | -46.623 | 46.454 | -68.997 |
| 45.177 | -44.979 | 46.572 | -26.205 |
| 45.210 | -58.563 | 47.201 | -51.417 |
| 45.254 | -15.586 | 47.616 | -58.709 |
| 45.276 | -29.659 | 47.942 | -64.643 |
| 45.282 | -34.283 | 47.966 | -75.439 |
| 45.360 | -36.108 | 48.034 | -67.316 |
| 45.368 | -43.676 | 48.276 | -44.155 |
| 45.467 | -42.801 | 48.595 | -60.845 |
| 45.471 | -59.235 | 48.651 | -17.411 |
| 45.509 | -65.259 | 48.663 | -46.911 |
| 45.669 | -82.410 | 48.724 | -52.067 |
| 45.847 | -45.211 | 48.774 | -70.729 |
| 45.980 | -44.608 | 48.781 | -47.063 |
| 46.024 | -40.249 | 48.867 | -68.442 |
| 46.104 | -0.7982 | 48.905 | -48.966 |
| 46.399 | -81.990 | 49.015 | -0.6115 |
| 46.449 | -32.692 | 49.196 | -51.811 |
| 46.573 | -50.843 | 49.216 | -45.817 |
| 46.694 | -52.115 | 49.229 | -49.482 |
| 46.707 | -39.481 | 49.484 | -52.820 |
| 46.754 | -53.170 | 49.587 | -46.449 |
| 46.807 | -43.575 | 49.698 | -72.013 |
| 47.134 | -62.988 | 49.769 | -82.436 |
| 47.216 | -47.819 | 49.963 | -93.514 |
| 47.393 | -68.130 | 50.001 | -62.086 |
| 47.573 | -35.036 | 50.216 | -46.291 |
| 47.888 | -54.818 | 50.332 | -32.836 |
| 47.991 | -0.6766 | 50.348 | -113.964 |
| 47.991 | -67.513 | 50.529 | 0.1864 |
| 48.175 | -17.844 | 50.535 | 33.788 |
| 48.177 | -0.7928 | 50.983 | 0.4551 |
| 48.250 | -35.227 | 51.114 | 0.0250 |
| 48.259 | -44.544 | 51.264 | 30.341 |
| 48.359 | -96.225 | 51.317 | -72.641 |
| 48.391 | -99.010 | 51.400 | -86.154 |
| 48.445 | -57.422 | 51.468 | 0.6028 |
| 48.571 | -98.686 | 51.588 | -77.819 |
| 48.630 | -66.496 | 51.618 | 10.308 |
| 48.877 | -54.514 | 51.717 | -14.958 |
| 49.004 | -40.455 | 51.736 | -94.612 |
| 49.298 | -68.190 | 51.854 | -0.1311 |
| 49.456 | -52.606 | 51.890 | 46.617 |
| 49.458 | -99.275 | 51.900 | -0.8099 |
| 49.493 | -56.859 | 51.998 | 32.688 |
| 49.727 | -59.576 | 52.001 | -37.671 |
| 49.800 | -32.061 | 52.003 | -31.879 |
| 49.856 | -32.500 | 52.005 | -37.897 |
| 49.860 | -29.212 | 52.014 | -54.880 |
| 50.018 | -62.268 | 52.043 | 22.898 |
| 50.028 | -41.380 | 52.275 | 41.691 |
| 50.092 | -58.433 | 52.503 | -115.106 |
| 50.200 | -54.118 | 52.597 | -16.726 |
| 50.202 | -52.337 | 52.753 | 13.417 |
| 50.208 | -58.284 | 52.777 | 0.9289 |
| 50.391 | -45.408 | 52.783 | -117.739 |
| 50.401 | -57.657 | 52.811 | -0.9121 |
| 50.470 | -42.500 | 53.103 | 0.2677 |
| 50.471 | -49.041 | 53.142 | 37.685 |
| 50.510 | -39.097 | 53.155 | -22.952 |
| 51.098 | -36.078 | 53.174 | -134.535 |
| 51.128 | -24.476 | 53.350 | -105.133 |
| 51.170 | -24.083 | 53.651 | 0.0947 |
| 51.528 | -55.802 | 53.675 | -45.573 |
| 51.602 | -39.668 | 53.818 | 39.849 |
| 51.642 | -11.243 | 53.836 | -59.566 |
| 51.674 | -32.491 | 53.861 | 19.898 |
| 51.705 | -34.543 | 53.893 | -0.7414 |
| 51.837 | -102.494 | 54.086 | -22.067 |
| 52.162 | -57.407 | 54.091 | -146.916 |
| 52.329 | -79.959 | 54.305 | -0.0901 |
| 52.515 | -40.937 | 54.318 | 15.069 |
| 52.543 | -62.504 | 54.508 | 16.434 |
| 52.626 | -52.892 | 54.525 | -75.393 |
| 52.633 | -88.538 | 54.847 | -54.213 |
| 52.670 | -39.500 | 54.980 | -0.1014 |
| 52.829 | -87.158 | 55.180 | 21.241 |
| 52.848 | -67.737 | 55.236 | 13.333 |
| 52.911 | -17.994 | 55.254 | -22.376 |
| 53.384 | -55.807 | 55.443 | -33.645 |
| 53.414 | -44.084 | 55.565 | 0.0687 |
| 53.425 | -34.825 | 55.571 | 0.6666 |
| 53.540 | -42.353 | 55.798 | 38.603 |
| 53.592 | -21.940 | 55.798 | 43.605 |
| 53.646 | -24.410 | 55.807 | -66.211 |
| 53.732 | -31.259 | 55.991 | -55.456 |
| 53.827 | -0.6508 | 56.084 | -30.973 |
| 54.122 | -73.682 | 56.178 | -78.579 |
| 54.238 | -60.693 | 56.251 | 0.0035 |
| 54.282 | -61.926 | 56.305 | 0.2937 |
| 54.431 | -22.652 | 56.322 | -96.450 |
| 54.437 | -43.474 | 56.622 | 34.281 |
| 54.552 | -42.957 | 56.635 | -37.900 |
| 54.778 | -68.704 | 56.680 | -58.309 |
| 54.930 | -18.554 | 56.743 | -11.794 |
| 54.994 | -29.082 | 56.839 | 16.843 |
| 55.094 | -33.163 | 56.843 | 37.366 |
| 55.115 | -95.481 | 56.851 | -72.894 |
| 55.124 | 0.0258 | 56.928 | -19.922 |
| 55.320 | -36.951 | 57.371 | -120.980 |
| 55.364 | -56.645 | 57.457 | 0.9582 |
| 55.568 | -20.951 | 57.479 | -24.373 |
| 55.650 | -0.4633 | 57.482 | -20.016 |
| 55.716 | -46.696 | 57.568 | -42.624 |
| 55.738 | -61.455 | 57.581 | -51.638 |
| 55.739 | -56.171 | 57.742 | -49.897 |
| 55.826 | -55.105 | 57.759 | 0.1306 |
| 55.874 | -55.902 | 57.804 | 0.0033 |
| 55.920 | -41.071 | 57.979 | -57.174 |
| 55.975 | -57.977 | 58.057 | -34.522 |
| 55.979 | -32.159 | 58.386 | -140.881 |
| 56.039 | -33.510 | 58.635 | -51.930 |
| 56.070 | -61.781 | 58.725 | 0.8485 |
| 56.282 | -18.539 | 58.783 | -10.917 |
| 56.329 | -35.193 | 58.976 | -10.647 |
| 56.455 | 18.834 | 59.016 | -45.855 |
| 56.491 | -34.299 | 59.062 | -57.064 |
| 56.614 | -62.185 | 59.141 | 0.0555 |
| 56.706 | -53.601 | 59.362 | -0.2443 |
| 56.748 | -73.581 | 59.512 | -0.2294 |
| 56.763 | -35.490 | 59.530 | -10.999 |
| 56.856 | 50.026 | 59.585 | 0.0199 |
| 56.885 | -28.441 | 59.796 | -37.654 |
| 56.947 | -35.835 | 59.803 | -50.015 |
| 57.319 | 0.2647 | 59.816 | -0.1389 |
| 57.353 | 0.6052 | 59.837 | -68.232 |
| 57.370 | -58.860 | 59.882 | 64.734 |
| 57.428 | -72.902 | 59.931 | 0.7967 |
| 57.479 | -20.552 | 60.241 | -64.975 |
| 57.494 | 0.2834 | 60.314 | -148.445 |
| 57.535 | -38.322 | 60.486 | -0.0437 |
| 57.628 | -54.329 | 60.527 | 0.3178 |
| 57.692 | -65.162 | 60.542 | -10.758 |
| 57.715 | -25.571 | 60.793 | -19.316 |
| 57.959 | -37.616 | 60.802 | 0.9188 |
| 58.038 | -24.513 | 60.839 | -0.2895 |
| 58.161 | -36.144 | 61.020 | -0.0009 |
| 58.189 | -69.833 | 61.237 | 23.329 |
| 58.315 | -21.505 | 61.306 | -0.5408 |
| 58.468 | 0.0962 | 61.516 | -19.798 |
| 58.509 | -34.051 | 61.582 | -0.0525 |
| 58.876 | 0.2464 | 61.602 | 0.0266 |
| 59.038 | -14.878 | 61.760 | -63.161 |
| 59.085 | -34.100 | 61.967 | -0.8666 |
| 59.227 | -68.953 | 61.970 | 0.2753 |
| 59.476 | -65.883 | 62.047 | -15.891 |
| 59.540 | -25.731 | 62.292 | -0.8430 |
| 59.652 | -18.532 | 62.570 | -10.742 |
| 59.696 | -42.421 | 62.572 | -12.445 |
| 59.966 | -46.336 | 62.856 | -69.574 |
| 60.007 | -0.5871 | 62.958 | 13.434 |
| 60.172 | -10.301 | 63.133 | 10.303 |
| 60.198 | -59.100 | 63.198 | 0.7026 |
| 60.245 | -43.619 | 63.266 | 29.734 |
| 60.335 | 0.7098 | 63.286 | 18.477 |
| 60.557 | 0.0266 | 63.325 | 24.125 |
| 60.647 | -28.251 | 63.545 | -12.172 |
| 60.811 | 0.9875 | 63.627 | 0.1943 |
| 61.210 | -16.005 | 63.789 | -18.958 |
| 61.214 | -11.654 | 63.912 | -16.282 |
| 61.306 | -28.148 | 63.912 | -26.088 |
| 61.339 | -33.049 | 63.976 | -46.714 |
| 61.358 | -51.124 | 63.989 | -37.951 |
| 61.543 | 0.6506 | 64.063 | -0.3604 |
| 61.550 | -10.507 | 64.377 | 69.085 |
| 61.594 | 37.132 | 64.402 | -15.472 |
| 61.673 | -0.1462 | 64.472 | 33.327 |
| 61.807 | -0.3132 | 64.854 | -0.2329 |
| 61.823 | 37.338 | 65.021 | 25.312 |
| 61.874 | -0.0643 | 65.321 | 0.9572 |
| 62.068 | -12.815 | 65.408 | -13.165 |
| 62.074 | -28.908 | 65.443 | 27.089 |
| 62.080 | 36.990 | 65.458 | 0.5627 |
| 62.162 | -41.293 | 65.474 | 25.835 |
| 62.270 | 0.8714 | 65.492 | 0.0464 |
| 62.287 | 0.2389 | 65.643 | 0.2000 |
| 62.361 | -49.170 | 65.887 | -0.5867 |
| 62.436 | -0.1784 | 65.946 | 98.367 |
| 62.480 | -48.622 | 66.033 | -15.420 |
| 62.577 | 12.140 | 66.109 | 110.818 |
| 62.747 | -0.8278 | 66.151 | 39.908 |
| 62.917 | -0.3799 | 66.160 | 34.183 |
| 62.971 | -24.709 | 66.191 | 16.887 |
| 62.973 | -0.3019 | 66.205 | 0.3701 |
| 63.025 | 19.095 | 66.276 | -44.918 |
| 63.143 | -27.913 | 66.408 | -19.743 |
| 63.476 | 0.2627 | 66.434 | 15.226 |
| 63.615 | 29.880 | 66.572 | 24.246 |
| 63.690 | -10.494 | 66.635 | -14.428 |
| 63.711 | 39.860 | 66.651 | -0.0785 |
| 63.962 | -41.127 | 66.762 | 14.783 |
| 64.206 | 0.5699 | 66.867 | 0.1879 |
| 64.451 | 13.129 | 66.909 | -0.9599 |
| 64.542 | 0.5171 | 66.951 | 77.764 |
| 64.732 | 34.283 | 66.964 | 21.625 |
| 64.777 | -19.322 | 67.020 | 43.384 |
| 64.778 | 42.222 | 67.131 | 52.463 |
| 64.863 | 0.1417 | 67.220 | 41.664 |
| 64.904 | -0.4593 | 67.253 | -10.475 |
| 64.978 | 10.184 | 67.297 | -31.288 |
| 65.198 | 17.690 | 67.427 | 19.218 |
| 65.272 | 63.813 | 67.489 | -0.8607 |
| 65.351 | 13.018 | 67.819 | 95.058 |
| 65.434 | 39.731 | 68.177 | 0.3742 |
| 65.563 | -18.514 | 68.317 | 38.605 |
| 65.843 | 59.599 | 68.380 | 55.379 |
| 65.919 | -57.573 | 68.384 | -0.1576 |
| 66.128 | 24.209 | 68.402 | 32.760 |
| 66.235 | 37.681 | 68.595 | 31.843 |
| 66.321 | 65.195 | 68.664 | -39.830 |
| 66.421 | 21.697 | 68.752 | -24.075 |
| 66.508 | 14.467 | 68.883 | 0.1293 |
| 66.634 | 63.928 | 68.912 | 0.2154 |
| 66.916 | 58.543 | 69.090 | -17.589 |
| 66.927 | 28.239 | 69.136 | 16.992 |
| 67.026 | 24.014 | 69.328 | -0.8838 |
| 67.125 | 47.475 | 69.358 | 73.458 |
| 67.229 | 35.387 | 69.457 | 40.526 |
| 67.528 | 20.582 | 69.465 | 101.094 |
| 67.541 | -0.1356 | 69.576 | 13.613 |
| 67.598 | 50.986 | 69.579 | -22.063 |
| 67.661 | 17.457 | 69.654 | -56.252 |
| 68.013 | 56.520 | 69.750 | 94.899 |
| 68.049 | 31.606 | 69.953 | 0.3519 |
| 68.226 | 51.030 | 70.111 | 123.617 |
| 68.267 | 36.847 | 70.120 | 0.0039 |
| 68.534 | -0.7159 | 70.122 | 34.431 |
| 68.586 | 41.852 | 70.125 | 187.912 |
| 68.709 | 0.3050 | 70.154 | 40.318 |
| 68.791 | 35.719 | 70.267 | 112.151 |
| 68.882 | 65.385 | 70.469 | -0.3569 |
| 68.985 | 16.677 | 70.473 | 160.836 |
| 69.031 | 70.120 | 70.636 | -87.163 |
| 69.413 | 13.743 | 70.755 | -94.489 |
| 69.492 | 36.185 | 70.972 | 27.264 |
| 69.668 | -0.4437 | 71.005 | 58.661 |
| 69.715 | 22.515 | 71.020 | 56.044 |
| 69.889 | 72.076 | 71.205 | 44.278 |
| 69.930 | 61.088 | 71.249 | 11.936 |
| 69.979 | 35.021 | 71.305 | -41.543 |
| 70.104 | 80.545 | 71.317 | 83.581 |
| 70.347 | 64.140 | 71.374 | -35.640 |
| 70.465 | 28.635 | 71.385 | 49.074 |
| 70.521 | 33.453 | 71.468 | 20.027 |
| 70.587 | 25.910 | 71.668 | 72.164 |
| 70.603 | 51.245 | 71.741 | 11.540 |
| 70.635 | 0.2962 | 71.762 | -0.6136 |
| 70.751 | 62.270 | 72.296 | 0.8656 |
| 70.754 | 15.816 | 72.314 | -0.0716 |
| 70.847 | 20.657 | 72.356 | 165.545 |
| 70.975 | 56.557 | 72.435 | 34.122 |
| 71.164 | 126.090 | 72.476 | -60.814 |
| 71.226 | 0.1760 | 72.603 | 56.160 |
| 71.264 | 32.024 | 72.640 | -49.068 |
| 71.400 | 90.847 | 72.681 | -14.065 |
| 71.460 | 108.587 | 72.721 | 0.4209 |
| 71.538 | 29.019 | 72.819 | 141.964 |
| 71.699 | 84.570 | 72.913 | 143.449 |
| 71.931 | 81.195 | 72.939 | 0.6939 |
| 71.944 | 29.356 | 72.998 | -60.812 |
| 71.960 | -0.3108 | 73.064 | 38.272 |
| 71.967 | 46.321 | 73.224 | -0.3574 |
| 72.053 | 47.098 | 73.256 | 107.854 |
| 72.081 | 63.955 | 73.344 | 26.951 |
| 72.125 | 34.306 | 73.353 | 37.053 |
| 72.127 | 76.358 | 73.358 | 53.237 |
| 72.146 | 89.233 | 73.368 | 155.196 |
| 72.343 | 0.6599 | 73.728 | -38.946 |
| 72.421 | 24.001 | 73.781 | 112.576 |
| 72.476 | 51.526 | 73.899 | -69.453 |
| 72.609 | -0.2375 | 73.921 | 96.731 |
| 72.917 | 79.678 | 74.117 | 24.603 |
| 73.021 | 28.324 | 74.254 | 159.989 |
| 73.035 | 16.660 | 74.298 | -12.440 |
| 73.056 | 19.230 | 74.384 | -50.446 |
| 73.151 | 0.2533 | 74.411 | -16.994 |
| 73.222 | 31.207 | 74.526 | 156.992 |
| 73.282 | 92.355 | 74.591 | 0.0799 |
| 73.294 | 40.580 | 74.600 | 93.521 |
| 73.711 | 37.737 | 74.656 | 59.512 |
| 73.750 | 37.889 | 74.705 | 26.851 |
| 73.855 | 71.349 | 74.855 | 136.585 |
| 73.863 | 134.754 | 74.862 | 0.2653 |
| 73.870 | 90.029 | 75.473 | 95.182 |
| 73.888 | 99.928 | 75.641 | -72.327 |
| 73.889 | 64.055 | 75.820 | -48.587 |
| 74.202 | 73.930 | 76.333 | 136.352 |
| 74.543 | 18.242 | 76.377 | 86.228 |
| 74.743 | 99.485 | 76.617 | 25.340 |
| 74.825 | 93.987 | 76.634 | -29.295 |
| 74.885 | 31.484 | 76.658 | -18.884 |
| 74.897 | 20.840 | 76.772 | 63.381 |
| 74.902 | 109.591 | 76.794 | -17.633 |
| 74.944 | 40.811 | 76.845 | -53.955 |
| 74.961 | 77.631 | 76.997 | 184.729 |
| 75.060 | 58.547 | 77.070 | 0.7727 |
| 75.071 | 57.930 | 77.206 | -33.178 |
| 75.072 | 112.078 | 77.219 | 121.625 |
| 75.115 | 76.928 | 77.388 | 73.387 |
| 75.127 | 68.942 | 77.596 | 31.316 |
| 75.216 | 0.7006 | 77.621 | 24.580 |
| 75.250 | 57.657 | 77.622 | 145.971 |
| 75.294 | 91.840 | 77.766 | 70.651 |
| 75.316 | 0.5877 | 77.823 | 101.279 |
| 75.360 | 60.088 | 77.832 | 139.265 |
| 75.396 | 44.431 | 77.856 | -99.637 |
| 75.502 | 0.4072 | 77.995 | 169.864 |
| 75.696 | 124.281 | 78.123 | -53.094 |
| 75.762 | 43.485 | 78.248 | 31.826 |
| 75.762 | 108.100 | 78.478 | 0.8765 |
| 76.145 | 88.411 | 78.490 | 55.412 |
| 76.178 | 108.729 | 78.650 | 73.858 |
| 76.201 | 114.190 | 78.683 | -82.283 |
| 76.202 | 98.818 | 78.842 | -0.5158 |
| 76.251 | 130.635 | 78.846 | -25.900 |
| 76.355 | 132.979 | 78.866 | 178.873 |
| 76.524 | 100.048 | 78.875 | 132.718 |
| 76.531 | 61.179 | 79.002 | 18.323 |
| 76.975 | 57.156 | 79.281 | 62.641 |
| 77.135 | 79.337 | 79.303 | 75.023 |
| 77.137 | 51.444 | 79.498 | 39.983 |
| 77.141 | 39.916 | 79.502 | -0.1980 |
| 77.223 | 86.164 | 79.638 | 15.408 |
| 77.231 | 89.416 | 79.756 | 104.015 |
| 77.442 | 40.205 | 79.977 | 93.619 |
| 77.567 | 140.673 | 80.068 | 55.024 |
| 77.589 | 168.966 | 80.282 | 48.153 |
| 77.716 | 55.393 | 80.324 | 117.728 |
| 77.851 | 18.234 | 80.336 | 94.677 |
| 78.028 | 79.203 | 80.382 | 88.612 |
| 78.055 | 127.750 | 80.643 | -0.7732 |
| 78.381 | 77.465 | 80.772 | 84.166 |
| 78.656 | 64.105 | 80.826 | 49.871 |
| 78.730 | 12.339 | 80.847 | 115.004 |
| 78.944 | 101.305 | 80.949 | 54.494 |
| 78.975 | 60.373 | 81.051 | 111.165 |
| 79.062 | 69.023 | 81.083 | 48.885 |
| 79.063 | 137.702 | 81.399 | 78.051 |
| 79.208 | 140.583 | 81.503 | 111.905 |
| 79.275 | 44.574 | 81.511 | 113.288 |
| 79.554 | 115.644 | 81.532 | 97.032 |
| 79.657 | 87.247 | 81.808 | 70.187 |
| 79.781 | 120.449 | 81.843 | 161.759 |
| 79.915 | 44.759 | 81.892 | 23.229 |
| 80.029 | 44.776 | 82.248 | 52.925 |
| 80.182 | 106.747 | 82.327 | 18.077 |
| 80.371 | 84.799 | 82.508 | 159.829 |
| 80.427 | 45.786 | 82.524 | 95.864 |
| 80.446 | 125.515 | 82.669 | 65.128 |
| 80.600 | 20.884 | 82.723 | 55.157 |
| 80.787 | 40.616 | 82.873 | 93.095 |
| 80.827 | 56.884 | 83.037 | 69.254 |
| 80.912 | 50.209 | 83.150 | 42.946 |
| 81.062 | 76.882 | 83.213 | 43.198 |
| 81.068 | 78.275 | 83.393 | 114.720 |
| 81.075 | 91.134 | 83.522 | 63.127 |
| 81.107 | -0.2145 | 83.820 | -0.7172 |
| 81.194 | 78.443 | 83.901 | 54.406 |
| 81.250 | 68.688 | 83.931 | 105.051 |
| 81.559 | 45.231 | 84.084 | 58.728 |
| 81.586 | 82.597 | 84.220 | 51.949 |
| 81.605 | 36.119 | 84.250 | 85.628 |
| 81.643 | 41.579 | 84.263 | 26.014 |
| 81.663 | 129.191 | 84.267 | 50.527 |
| 81.767 | 49.802 | 84.328 | 59.089 |
| 81.782 | 53.509 | 84.452 | 137.987 |
| 81.876 | 81.891 | 84.628 | 39.928 |
| 82.019 | 126.699 | 84.765 | 20.385 |
| 82.029 | 86.773 | 84.789 | 64.979 |
| 82.197 | 86.306 | 84.792 | 61.866 |
| 82.603 | 71.878 | 84.804 | 61.845 |
| 82.841 | 148.977 | 84.856 | 97.470 |
| 82.896 | 120.744 | 84.875 | 49.862 |
| 82.952 | 104.734 | 84.964 | 124.363 |
| 82.977 | 61.007 | 85.046 | 65.975 |
| 83.260 | 74.289 | 85.165 | 117.855 |
| 83.348 | 53.629 | 85.262 | 67.260 |
| 83.384 | 99.961 | 85.353 | 68.501 |
| 83.530 | 108.077 | 85.433 | -39.161 |
| 83.729 | 83.591 | 85.751 | 92.114 |
| 83.947 | 112.510 | 85.788 | 30.697 |
| 83.975 | 15.668 | 85.795 | 99.813 |
| 83.977 | 81.381 | 86.001 | 24.004 |
| 84.039 | 105.832 | 86.228 | 105.173 |
| 84.053 | 33.094 | 86.242 | 83.224 |
| 84.062 | 121.646 | 86.272 | 0.4939 |
| 84.169 | 57.355 | 87.130 | 34.045 |
| 84.217 | 100.516 | 87.297 | 55.824 |
| 84.218 | 70.383 | 87.715 | 81.341 |
| 84.562 | 27.939 | 87.742 | -26.688 |
| 84.608 | 49.067 | 87.802 | 114.641 |
| 84.679 | 86.817 | 87.889 | 22.763 |
| 84.786 | 97.563 | 87.937 | 60.073 |
| 84.969 | 81.088 | 87.950 | 0.9942 |
| 85.013 | 64.651 | 88.150 | 65.413 |
| 85.031 | 99.618 | 88.183 | 23.146 |
| 85.074 | 49.241 | 88.235 | 85.170 |
| 85.141 | 76.897 | 88.261 | 26.516 |
| 85.203 | 47.917 | 88.285 | 62.826 |
| 85.228 | 97.442 | 88.375 | 58.896 |
| 85.396 | 89.170 | 88.480 | 55.169 |
| 85.601 | 157.912 | 88.480 | 83.896 |
| 85.704 | 16.823 | 88.604 | 63.370 |
| 85.708 | 17.126 | 88.707 | -0.3400 |
| 85.804 | 102.301 | 88.720 | 78.504 |
| 86.250 | 22.578 | 88.782 | 88.198 |
| 86.314 | 81.540 | 88.798 | 27.891 |
| 86.323 | 44.448 | 89.257 | 10.283 |
| 86.493 | 59.588 | 89.264 | 75.278 |
| 86.495 | 30.437 | 89.586 | 30.472 |
| 86.717 | 20.386 | 89.661 | 59.497 |
| 86.936 | 91.496 | 89.728 | 15.824 |
| 87.003 | 88.361 | 89.805 | 25.315 |
| 87.069 | 107.855 | 89.832 | -30.275 |
| 87.167 | 95.000 | 89.909 | 75.147 |
| 87.184 | 34.691 | 90.030 | 21.646 |
| 87.280 | 61.434 | 90.055 | 76.525 |
| 87.291 | 64.900 | 90.072 | 25.980 |
| 87.385 | 18.800 | 90.207 | 12.953 |
| 87.436 | 87.474 | 90.558 | 115.284 |
| 87.743 | 59.596 | 90.627 | 71.210 |
| 87.770 | 76.203 | 90.640 | -11.680 |
| 87.879 | 54.948 | 90.660 | 30.163 |
| 87.893 | 66.456 | 90.669 | 59.032 |
| 88.224 | 48.371 | 90.697 | 29.462 |
| 88.310 | 108.199 | 90.724 | -17.038 |
| 88.418 | 56.007 | 90.732 | 64.180 |
| 88.523 | 25.840 | 90.903 | 40.365 |
| 88.857 | 19.960 | 91.050 | 0.4777 |
| 89.000 | 17.394 | 91.137 | -21.555 |
| 89.190 | 46.977 | 91.171 | 42.680 |
| 89.252 | 13.452 | 91.179 | 36.468 |
| 89.322 | 53.838 | 91.231 | 21.737 |
| 89.760 | 54.924 | 91.296 | -12.393 |
| 89.837 | 52.846 | 91.297 | -12.768 |
| 89.951 | 78.086 | 91.378 | 42.930 |
| 89.966 | 38.666 | 91.625 | -13.388 |
| 90.309 | 71.501 | 91.654 | -12.107 |
| 90.470 | 33.219 | 91.665 | -12.235 |
| 90.626 | 10.370 | 91.685 | -14.104 |
| 90.650 | -10.918 | 91.959 | 19.864 |
| 90.695 | 11.976 | 92.056 | 93.341 |
| 90.838 | -16.455 | 92.121 | -0.8495 |
| 90.869 | -0.5745 | 92.211 | 82.666 |
| 91.044 | 21.338 | 92.252 | 50.758 |
| 91.212 | 25.859 | 92.256 | -12.324 |
| 91.220 | 30.841 | 92.359 | -50.208 |
| 91.310 | 80.507 | 92.391 | -14.144 |
| 91.376 | 71.870 | 92.625 | -43.816 |
| 91.444 | 27.651 | 92.665 | 33.286 |
| 91.511 | 0.5221 | 92.677 | 22.089 |
| 91.882 | 61.225 | 92.756 | 12.891 |
| 92.324 | 44.124 | 92.835 | -32.801 |
| 92.335 | 34.835 | 93.085 | -36.797 |
| 92.340 | 11.117 | 93.123 | -14.521 |
| 92.356 | -0.8843 | 93.219 | 44.134 |
| 92.390 | 81.466 | 93.253 | 41.493 |
| 92.484 | -22.626 | 93.270 | 60.308 |
| 92.580 | 28.544 | 93.360 | 72.215 |
| 92.630 | -25.527 | 93.453 | 34.563 |
| 92.650 | 37.487 | 93.589 | 29.226 |
| 92.694 | 60.887 | 93.610 | 59.073 |
| 92.886 | 57.115 | 93.726 | 0.0582 |
| 93.012 | -41.880 | 93.778 | 42.119 |
| 93.295 | -29.307 | 93.790 | 27.117 |
| 93.444 | -54.827 | 93.806 | 56.732 |
| 93.631 | 39.716 | 94.097 | -30.705 |
| 93.743 | -26.521 | 94.196 | -61.710 |
| 93.791 | 62.501 | 94.218 | -0.0612 |
| 93.985 | 11.946 | 94.265 | -44.403 |
| 94.026 | -20.467 | 94.712 | 65.542 |
| 94.066 | 41.126 | 94.773 | -0.4983 |
| 94.351 | 68.541 | 95.002 | 0.6670 |
| 94.474 | -66.081 | 95.390 | -45.759 |
| 94.725 | 15.464 | 95.592 | -25.198 |
| 94.895 | 69.594 | 95.730 | 29.003 |
| 95.004 | 45.647 | 95.730 | -76.892 |
| 95.156 | 14.230 | 95.782 | -31.167 |
| 95.306 | -17.811 | 95.792 | -28.148 |
| 95.450 | -59.224 | 95.820 | 62.859 |
| 95.649 | 19.328 | 95.881 | 0.0416 |
| 96.003 | -33.716 | 95.953 | -79.764 |
| 96.041 | -0.2610 | 95.995 | -35.406 |
| 96.067 | 14.486 | 96.136 | 29.887 |
| 96.092 | 0.1877 | 96.163 | -10.215 |
| 96.112 | -18.282 | 96.204 | -17.100 |
| 96.261 | 21.479 | 96.266 | -11.652 |
| 96.341 | -25.947 | 96.294 | -10.427 |
| 96.390 | -0.1973 | 96.384 | -37.098 |
| 96.419 | -53.452 | 96.574 | 57.931 |
| 96.428 | -18.308 | 96.808 | -20.879 |
| 96.463 | -52.101 | 96.841 | 0.2680 |
| 96.505 | -11.240 | 97.086 | 56.166 |
| 96.512 | -16.858 | 97.305 | -35.858 |
| 96.544 | -15.909 | 97.468 | 0.6979 |
| 96.652 | -34.026 | 97.522 | -49.140 |
| 96.874 | -47.964 | 97.743 | -55.899 |
| 96.966 | -18.799 | 97.768 | -44.319 |
| 96.980 | -42.341 | 97.875 | -23.684 |
| 97.114 | -34.314 | 98.126 | -157.314 |
| 97.252 | -87.974 | 98.156 | -65.871 |
| 97.290 | -36.907 | 98.161 | -61.664 |
| 97.309 | -56.615 | 98.307 | -60.491 |
| 97.657 | 19.260 | 98.319 | -36.094 |
| 97.668 | 0.2847 | 98.571 | 53.534 |
| 98.174 | 0.6976 | 98.582 | -92.926 |
| 98.191 | -62.084 | 98.662 | -51.387 |
| 98.217 | -120.868 | 98.671 | -82.902 |
| 98.325 | -40.754 | 98.672 | -40.200 |
| 98.363 | -23.859 | 98.947 | -66.862 |
| 98.428 | -41.979 | 99.180 | -51.329 |
| 98.668 | 26.898 | 99.234 | -76.934 |
| 98.798 | -51.072 | 99.290 | -0.4533 |
| 98.832 | 37.644 | 99.337 | -28.556 |
| 98.863 | 0.6926 | 99.343 | -68.825 |
| 99.052 | -13.048 | 99.405 | -41.157 |
| 99.115 | 0.9025 | 99.442 | 26.107 |
| 99.127 | -34.362 | 99.465 | -86.530 |
| 99.173 | -44.931 | 99.530 | 0.2266 |
| 99.239 | -93.223 | 99.534 | -16.134 |
| 99.416 | 0.3686 | 99.609 | -42.855 |
| 99.723 | -68.896 | 99.661 | -17.135 |
| 99.821 | -194.772 | 99.740 | -0.9742 |
| 99.943 | 41.316 | 99.886 | -139.494 |
| 99.952 | -30.715 | 99.983 | -113.288 |
| 100.245 | -142.104 | 100.452 | -67.802 |
| 100.296 | -33.596 | 100.466 | 0.1298 |
| 100.710 | -61.596 | 100.628 | -96.500 |
| 100.954 | -67.452 | 101.139 | -52.245 |
| 101.460 | -68.093 | 101.172 | -64.647 |
| 101.603 | -79.815 | 101.239 | -23.095 |
| 102.280 | -109.246 | 101.299 | -95.746 |
| 102.284 | -98.265 | 101.300 | 11.214 |
| 102.310 | -79.856 | 101.770 | -121.571 |
| 103.293 | -192.119 | 101.850 | -68.074 |
| 103.456 | -116.712 | 102.062 | -49.859 |
| 103.458 | -54.145 | 102.229 | -77.702 |
| 103.487 | -84.491 | 102.306 | -101.243 |
| 103.728 | -77.322 | 102.968 | -85.786 |
| 103.904 | -108.341 | 103.541 | -105.780 |
| 104.079 | -45.033 | 103.573 | -101.095 |
| 104.146 | -153.146 | 103.842 | -49.565 |
| 104.413 | -50.315 | 104.281 | -42.714 |
| 104.654 | -124.096 | 104.540 | -89.586 |
| 105.507 | 11.833 | 105.084 | -173.185 |
| 105.517 | -79.937 | 105.106 | 0.2908 |
| 105.683 | -67.017 | 105.150 | -115.452 |
| 106.296 | -168.769 | 105.398 | -77.217 |
| 106.397 | -120.817 | 105.658 | -123.850 |
| 106.558 | -74.206 | 105.925 | -106.000 |
| 107.163 | -84.790 | 106.051 | -112.926 |
| 107.427 | -94.971 | 106.169 | -129.282 |
| 107.927 | -126.762 | 106.524 | -175.899 |
| 107.930 | -144.944 | 106.737 | -179.325 |
| 108.493 | -119.108 | 106.842 | -83.007 |
| 108.567 | -149.952 | 106.972 | -75.883 |
| 108.607 | -166.796 | 107.119 | -51.255 |
| 108.772 | -201.787 | 107.497 | -101.160 |
| 109.298 | -57.359 | 107.731 | -136.330 |
| 109.765 | -69.497 | 107.933 | -115.352 |
| 109.766 | -78.564 | 108.471 | -165.083 |
| 110.660 | -140.312 | 108.764 | -109.172 |
| 111.173 | -108.501 | 108.960 | -155.344 |
| 111.191 | -66.365 | 109.317 | -125.267 |
| 111.363 | -90.775 | 109.505 | -145.688 |
| 111.942 | -168.244 | 109.516 | -71.813 |
| 111.995 | -97.033 | 109.720 | -36.936 |
| 112.268 | -75.306 | 109.931 | -148.245 |
| 112.609 | -59.410 | 110.235 | -151.150 |
| 113.128 | -151.957 | 110.538 | -172.750 |
| 113.377 | -124.046 | 110.682 | -65.800 |
| 113.458 | -44.336 | 110.822 | -122.432 |
| 113.576 | -94.285 | 110.892 | -157.721 |
| 113.718 | -96.620 | 111.157 | -61.154 |
| 113.846 | -102.913 | 111.408 | -152.471 |
| 114.973 | -155.820 | 111.763 | -229.959 |
| 115.106 | -173.017 | 111.849 | -100.835 |
| 115.127 | -122.698 | 111.999 | -134.998 |
| 115.194 | -71.719 | 112.443 | -167.748 |
| 115.204 | -144.060 | 112.524 | -146.838 |
| 115.213 | -72.914 | 112.659 | -82.143 |
| 115.670 | -60.851 | 112.814 | -80.344 |
| 115.984 | -47.378 | 112.867 | -128.687 |
| 116.395 | -72.097 | 113.497 | -107.549 |
| 116.644 | -0.9305 | 113.662 | -61.222 |
| 117.488 | -105.738 | 114.160 | -63.145 |
| 117.566 | -92.609 | 114.358 | -92.805 |
| 117.969 | -125.496 | 114.747 | -97.519 |
| 118.260 | -114.902 | 115.233 | -144.589 |
| 118.464 | -22.452 | 115.423 | -99.688 |
| 118.690 | -33.137 | 115.425 | -101.223 |
| 118.821 | -33.558 | 115.548 | -132.984 |
| 119.229 | -217.597 | 115.568 | -105.530 |
| 119.290 | -170.355 | 115.970 | -72.452 |
| 119.325 | -27.738 | 116.011 | 22.350 |
| 119.764 | -111.487 | 116.158 | -139.657 |
| 120.093 | -19.615 | 116.262 | -84.663 |
| 120.458 | -26.882 | 116.295 | -58.724 |
| 121.159 | -92.913 | 116.495 | -81.068 |
| 121.481 | -128.278 | 116.497 | -131.461 |
| 121.690 | -96.582 | 116.545 | -41.165 |
| 121.940 | -81.863 | 116.695 | 0.5724 |
| 122.075 | -34.853 | 116.759 | -95.980 |
| 122.172 | -76.505 | 116.825 | -143.509 |
| 122.379 | -0.2668 | 116.888 | -56.703 |
| 122.454 | -20.254 | 116.960 | -0.9323 |
| 122.663 | -87.207 | 117.343 | -129.664 |
| 122.693 | 0.9853 | 117.445 | -62.342 |
| 123.160 | -28.470 | 117.484 | -109.744 |
| 123.165 | 69.623 | 118.136 | -21.580 |
| 123.223 | -20.023 | 118.201 | -54.141 |
| 123.396 | -42.201 | 118.377 | -25.674 |
| 123.562 | -85.319 | 118.379 | -191.196 |
| 123.596 | 24.415 | 118.684 | -178.599 |
| 123.928 | 0.1396 | 118.979 | -30.552 |
| 124.148 | -15.533 | 119.377 | -0.3278 |
| 124.287 | -35.919 | 119.494 | -113.786 |
| 124.485 | -71.546 | 119.811 | -95.757 |
| 124.690 | -29.123 | 120.054 | -84.579 |
| 124.852 | -0.0162 | 120.230 | -76.247 |
| 124.914 | -93.923 | 120.498 | -124.902 |
| 125.201 | -70.384 | 121.292 | -69.755 |
| 126.232 | -64.087 | 121.311 | -0.8460 |
| 126.632 | 22.145 | 121.726 | -49.134 |
| 126.655 | -73.451 | 121.791 | -0.7073 |
| 127.091 | 39.079 | 122.073 | 0.7960 |
| 127.902 | 42.347 | 122.158 | -111.819 |
| 127.971 | -0.8859 | 122.835 | 0.1393 |
| 128.114 | -23.581 | 123.012 | -82.348 |
| 128.282 | 33.131 | 123.224 | 97.223 |
| 128.423 | -57.927 | 123.354 | -89.196 |
| 129.268 | 0.5046 | 124.130 | -43.179 |
| 129.325 | -0.6999 | 124.900 | 0.4570 |
| 129.464 | 50.708 | 124.928 | -59.820 |
| 129.471 | 14.045 | 125.014 | -0.6035 |
| 129.864 | -17.517 | 125.100 | 52.204 |
| 130.211 | -15.110 | 125.196 | 0.4051 |
| 130.654 | 25.603 | 125.235 | 0.4421 |
| 130.832 | 106.454 | 125.564 | 12.855 |
| 130.848 | -10.868 | 126.414 | -74.156 |
| 131.103 | -0.2016 | 126.692 | -20.177 |
| 131.314 | -0.0897 | 126.823 | 61.002 |
| 132.168 | 207.191 | 126.866 | 58.634 |
| 132.680 | 56.395 | 126.914 | -12.471 |
| 132.793 | -0.4515 | 127.219 | 0.2351 |
| 133.086 | 59.978 | 127.465 | 22.709 |
| 133.896 | 132.022 | 127.869 | -21.265 |
| 134.058 | 136.972 | 127.960 | 12.727 |
| 134.127 | 0.6847 | 128.677 | 30.616 |
| 134.489 | 80.375 | 128.714 | 96.660 |
| 134.509 | 134.411 | 128.747 | 134.139 |
| 134.635 | 137.493 | 129.848 | 38.560 |
| 134.898 | 56.467 | 129.983 | -31.166 |
| 135.149 | 125.499 | 130.086 | 77.379 |
| 135.258 | 169.917 | 130.324 | 106.873 |
| 135.346 | 127.234 | 130.405 | 71.924 |
| 135.445 | 111.038 | 130.775 | 116.660 |
| 135.573 | 57.345 | 131.487 | 24.325 |
| 135.930 | 203.043 | 131.845 | 121.979 |
| 136.105 | 193.217 | 132.717 | 40.727 |
| 136.147 | 140.703 | 132.922 | 153.507 |
| 136.674 | 85.214 | 133.771 | 129.502 |
| 136.912 | 184.127 | 134.025 | 47.326 |
| 137.022 | -0.5819 | 134.133 | 29.155 |
| 137.659 | 245.598 | 134.293 | 60.728 |
| 137.724 | 132.582 | 134.965 | -57.818 |
| 137.775 | 122.627 | 135.174 | 46.586 |
| 138.191 | 226.168 | 135.233 | 82.816 |
| 138.239 | 185.403 | 135.261 | 70.050 |
| 138.290 | 207.691 | 135.424 | 56.354 |
| 138.374 | 143.360 | 135.730 | 98.052 |
| 138.605 | 238.522 | 135.980 | 193.827 |
| 138.711 | 74.096 | 135.983 | 27.578 |
| 138.859 | 185.749 | 136.040 | 211.599 |
| 139.125 | 136.523 | 136.063 | 142.740 |
| 139.174 | 52.748 | 136.171 | 107.108 |
| 139.368 | 133.077 | 136.238 | 28.666 |
| 139.425 | 164.644 | 136.517 | 86.677 |
| 139.588 | 30.668 | 136.528 | 84.997 |
| 139.621 | 215.767 | 136.575 | 80.476 |
| 140.116 | 38.208 | 136.931 | 144.198 |
| 140.143 | 94.739 | 136.944 | 92.317 |
| 140.171 | 175.958 | 137.101 | 169.091 |
| 140.501 | 85.375 | 137.412 | 92.841 |
| 140.764 | 103.565 | 137.558 | 160.684 |
| 140.771 | 90.267 | 137.608 | 139.252 |
| 141.111 | 131.673 | 137.881 | 0.4123 |
| 141.348 | 253.931 | 138.568 | 244.517 |
| 141.496 | 174.673 | 138.568 | 190.698 |
| 141.633 | 103.884 | 138.615 | -36.430 |
| 141.771 | 155.528 | 139.014 | 101.689 |
| 142.122 | 74.148 | 139.958 | 55.919 |
| 142.162 | 190.523 | 140.456 | 238.859 |
| 142.514 | 165.376 | 140.891 | 252.947 |
| 142.655 | 88.179 | 141.301 | 117.420 |
| 142.791 | 109.063 | 141.476 | 74.142 |
| 142.807 | 246.792 | 141.489 | 153.385 |
| 142.993 | 97.423 | 141.765 | 73.345 |
| 143.010 | 156.201 | 141.864 | 278.179 |
| 143.079 | 174.666 | 142.019 | 110.694 |
| 143.875 | 197.360 | 142.027 | 165.776 |
| 144.626 | 141.009 | 142.339 | 104.610 |
| 144.762 | 97.430 | 142.515 | 167.286 |
| 145.309 | 235.065 | 143.468 | 149.915 |
| 145.467 | 250.347 | 143.535 | 49.105 |
| 145.497 | 193.906 | 143.718 | 140.702 |
| 145.625 | 219.359 | 143.872 | 218.380 |
| 145.679 | 137.839 | 143.940 | 132.607 |
| 145.736 | 194.734 | 144.155 | 262.367 |
| 145.775 | 164.060 | 144.626 | 195.200 |
| 145.921 | 121.400 | 145.313 | 192.228 |
| 145.954 | 129.417 | 145.347 | 155.160 |
| 145.970 | 27.478 | 145.655 | 195.352 |
| 146.820 | 55.693 | 146.325 | 190.835 |
| 147.391 | 128.229 | 146.506 | 184.470 |
| 147.423 | 77.147 | 147.237 | 103.544 |
| 147.635 | 36.342 | 147.824 | 121.922 |
| 147.847 | 92.830 | 148.020 | 236.772 |
| 148.158 | 140.422 | 148.210 | 192.956 |
| 148.167 | 132.930 | 148.326 | 15.942 |
| 148.471 | 124.243 | 148.557 | 58.801 |
| 149.066 | 37.638 | 148.626 | 185.306 |
| 149.560 | 180.934 | 148.655 | 108.198 |
| 149.639 | 200.575 | 149.334 | 133.506 |
| 149.936 | 70.906 | 149.587 | 119.204 |
