# Supplementary material for: Prospect certainty for data-driven models
Source: Sci Rep. 2025 Mar 10;15:8278. doi: 10.1038/s41598-025-89679-6 (PMC11893788; doi:10.1038/s41598-025-89679-6)
Supplement: Supplementary file 2 — Supplementary Information 2. [file 41598_2025_89679_MOESM2_ESM.docx]

Supplementary Material

Paper Title *Prospect certainty for data-driven models*

Qais Yousef ^1,^* & Pu Li ^2,^*

1 ORCID: [0000-0003-0239-9150].

2 ORCID: [0000-0001-6481-9961].

1,2 Group of Process Optimization, Institute for Automation and Systems Engineering, Technische Universität Ilmenau, P.O. Box 100565, 98684 Ilmenau, Germany

* Correspondence: {qais.yousef, pu.li}@tu-ilmenau.de

**Theoretical Analyses Supporting Weighted Probability Compliance**

The following propositions are used to prove the satisfaction of (4) and (5) to the requirements of the weighted probability.

**Proposition 1**: If the standard deviation of the masks is lower than the distance between the logit and the mean of these masks, the weights of the masks will be higher than the weight of the logit.

**Proposition 2**: The weights for each sample in the group reflect its distance from the mean.

**Proposition 3**: If the logit equals the mean and matches any sample value, its weight will be only slightly greater than the sample's weight.

To prove the propositions, ensuring that the weights for each logit with its masks sum to one, let us assume that $\mu$ and $\sigma$ are the mean and the standard deviations of the samples, respectively.

Recall from (4) and (5) that the weights for each logit and each of its masks are formulated, respectively, as,

$\mathcal{w}_{i,t}=\frac{1}{\ln\left( \left| \hat{u}_{i,t}-\mu\right|+e+\epsilon\right)}$ (4)

$\mathcal{w}_{i,j,t}= \frac{1}{\ln\left( \left| \hat{u}_{i,j,t}-\mu\right|+e+\epsilon+s \right)}$ (5)

**Proof 1**:

If $\sigma<\left| \hat{u}_{i,t}-\mu\right|$, the weights for the masks are calculated using (5). Since $\ln\left( \left| \hat{u}_{i,j,t}-\mu\right|+e+\epsilon+s \right)$, will be smaller for values closer to the man, i.e. smaller distances, the formulation (5) will be larger. Given that the weights for the logits are identified using (4), $\ln\left( \left| \hat{u}_{i,t}-\mu\right|+e+\epsilon\right)$ will be smaller, allowing (4) to be larger. Hence, the weights of the masks $\mathcal{w}_{i,j,t}$ becomes larger than that of the logit $\mathcal{w}_{i,t}$, thus satisfying proposition 1 $∎$.

**Proof 2**:

Assuming that the distance $\left| \hat{u}_{i,j,t}-\mu\right|$ in (5) increases, then the denominator $\ln\left( \left| \hat{u}_{i,j,t}-\mu\right|+e+\epsilon+s \right)$ increases, making $\mathcal{w}_{i,j,t}$ decreases. It means that the masks far from the mean have lower weights. The opposite if $\left| \hat{u}_{i,j,t}-\mu\right|$ decreases, is also true. The same applies to equation (4), which satisfies proposition 2 $∎$.

**Proof 3**:

Let us assume that a node and its masks are located on the mean on the masks, i.e. $\hat{u}_{i,t}=\left\{ \hat{u}_{i,j,t} \right\}_{j=1}^{N_{i}^{M}}=\mu$. Substituting these values in (4) and (5), respectively, gives,

$$\mathcal{w}_{i,t}=\frac{1}{\ln\left( e+\epsilon\right)}$$

$$\mathcal{w}_{i,j,t}= \frac{1}{\ln\left( e+\epsilon+s \right)}$$

Given that $s$ is a very small constant, then $\ln\left( e+\epsilon\right)<\ln\left( e+\epsilon+s \right)$, which implies, $\mathcal{w}_{i,t}>\mathcal{w}_{i,j,t}$, suggesting that the logit will have slightly more influence over the masks, even if they have the same distance to the mean, thus satisfying proposition 3 $∎$.
